# Learning multi-site harmonization of magnetic resonance images without traveling human phantoms

**DOI:** 10.1038/s44172-023-00140-w

**Published:** 2024-01-05

**Authors:** Siyuan Liu, Pew-Thian Yap

**Affiliations:** Department of Radiology and Biomedical Research Imaging Center (BRIC), University of North Carolina at Chapel Hill, Chapel Hill, NC USA

**Keywords:** Image processing, Biomedical engineering, Biomedical engineering, Imaging techniques

## Abstract

Harmonization improves Magn. Reson. Imaging (MRI) data consistency and is central to effective integration of diverse imaging data acquired across multiple sites. Recent deep learning techniques for harmonization are predominantly supervised in nature and hence require imaging data of the same human subjects to be acquired at multiple sites. Data collection as such requires the human subjects to travel across sites and is hence challenging, costly, and impractical, more so when sufficient sample size is needed for reliable network training. Here we show how harmonization can be achieved with a deep neural network that does not rely on traveling human phantom data. Our method disentangles site-specific appearance information and site-invariant anatomical information from images acquired at multiple sites and then employs the disentangled information to generate the image of each subject for any target site. We demonstrate with more than 6,000 multi-site T1- and T2-weighted images that our method is remarkably effective in generating images with realistic site-specific appearances without altering anatomical details. Our method allows retrospective harmonization of data in a wide range of existing modern large-scale imaging studies, conducted via different scanners and protocols, without additional data collection.

## Abstract (web summary)

Data harmonization of MRI scans can improve consistency when analysing MRI data from different instruments in different locations. Liu and Yap report an efficient deep neural network method to disentangle site-specific information from site-invariant anatomical information in MRI images. The approach allows data in a wide range of existing studies, conducted via different imaging protocols, to be harmonized.

## Introduction

Magnetic resonance imaging (MRI) is a non-invasive and versatile technique that provides good soft tissue contrasts useful for diagnosis, prognosis, and treatment monitoring. Since MRI experiments are costly and time-consuming, modern large-scale MRI studies typically rely on multiple imaging sites to collaboratively collect data with greater sample sizes for more comprehensive coverage of factors that can affect study outcomes, such as age, gender, geography, socioeconomic status, and disease subtypes. Notable examples of multi-site studies include the Adolescent Brain Cognitive Development (ABCD)^1^, the Alzheimer’s Disease Neuroimaging Initiative (ADNI)^2^, and the Australian Imaging, Biomarkers and Lifestyle Flagship Study of Aging (AIBL)^3^.

Multi-site data collection inevitably leads to an undesirable elevation of non-biological variability introduced by differences in scanners^4^ and imaging protocols^5^. Protocols can be prospectively harmonized by selecting the acquisition parameters that result in images with maximal inter-site consistency for the individual sites. However, prospective harmonization requires extensive data acquisition for parameter tuning, needs to be performed before each study, and does not allow for correction of data collected in studies that have already taken place. Moreover, significant inter-site variability can still occur in data collected with harmonized acquisition protocols simply due to irreconcilable differences between scanners^4^. Retrospective MRI harmonization^6^ overcomes these limitations by performing post-acquisition correction and is hence applicable to existing studies for improving the accuracy, reliability, and reproducibility of downstream processing, analyses, and inferences.

Existing retrospective harmonization methods are either statistics-based or learning-based (Supplementary Table 1). Statistics-based methods align intensity distributions via intensity normalization^7–11^ or batch effect adjustment^12–14^. However, these methods are typically limited to whole-image, but not detail-specific, harmonization.

Learning-based methods translate images between sites via nonlinear mappings determined using machine learning^15,16^ or deep learning^17–19^, with or without supervision. Machine learning methods predict harmonized images using regression models learned typically with supervision and with hand-crafted features. In contrast, deep learning techniques automatically extract features pertinent to the harmonization task. Supervised methods typically require training data acquired from traveling human phantoms, which might not always be available for large-scale, multi-site, or longitudinal studies. Unsupervised deep learning techniques^20–22^ determine mappings between sites using unpaired images and therefore avoid the need for traveling human phantom data. These methods are, however, unscalable to large-scale multi-site MRI harmonization as they typically learn pair-wise mappings between sites. For *N* sites, these methods learn *N*(*N* − 1) mappings for all-site pairs and therefore require a large amount of data for learning the multitude of network parameters. These pair-wise methods are also ineffective by not fully and jointly utilizing complementary information available from all sites.

**Fig. 1:**
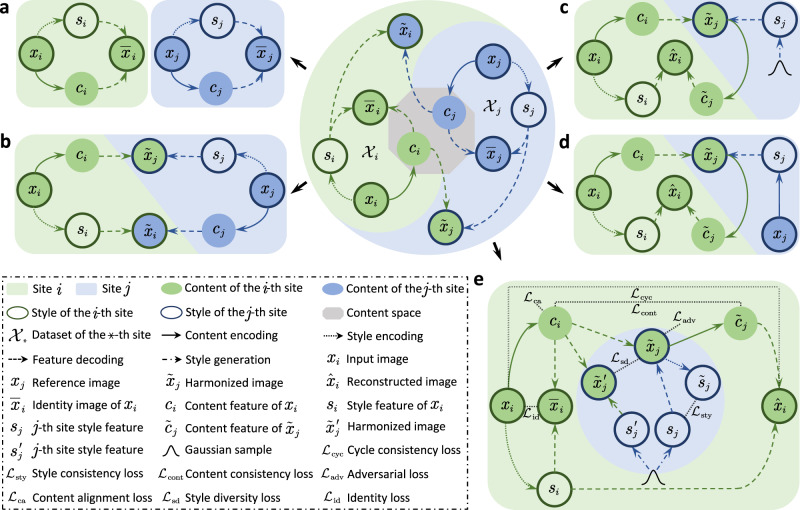
Overview of the multi-site unsupervised representation disentangler (MURD). **a** Representation disentanglement and combination. Given *n* images ${\{{x}_{i}\}}_{i = 1}^{n}$ from *N* sites ${\{{{{{{{{{\mathcal{X}}}}}}}}}_{i}\}}_{i = 1}^{N}$, each image *x*_*i*_ can be disentangled into a site-invariant content representation *c*_*i*_ and a site-specific style representation *s*_*i*_, and the disentangled content and style representations can be combined to reconstruct image ${\bar{x}}_{i}$. Similarly, an image *x*_*j*_ (*j* ≠ *i*) from a site ${{{{{{{{\mathcal{X}}}}}}}}}_{j}$ can be disentangled into content representation *c*_*j*_ and style representation *s*_*j*_ and then combined to reconstruct ${\bar{x}}_{j}$. **b** Representation disentanglement based harmonization. Style representations alter while content representations maintain during harmonization such that the *j*-site-harmonized image ${\tilde{x}}_{j}$ of *x*_*i*_ can be generated by combining the content representation *c*_*i*_ from *x*_*i*_ and style representation *s*_*j*_ from *x*_*j*_. Similarly, the *i*-site-harmonized image ${\tilde{x}}_{i}$ of *x*_*j*_ can be generated by *c*_*j*_ and *s*_*i*_. Representation disentanglement is achieved using content-style disentangled cycle translation (CS-DCT). **c** Site-specific CS-DCT. An image from the *i*th site *x*_*i*_ is encoded via content features *c*_*i*_ and style features *s*_*i*_. Style features of the *j*-th site *s*_*j*_ (*j* ≠ *i*) are generated using style generator ${G}_{j}^{{{{{{{{\rm{S}}}}}}}}}$. *s*_*j*_ and *c*_*i*_ are decoded to generate a harmonized image for the *j*th site ${\tilde{x}}_{j}$, which is in turn encoded to generate content features ${\tilde{c}}_{j}$ for reconstructing image ${\hat{x}}_{i}$ with style features *s*_*i*_. **d** Reference-specific CS-DCT. Unlike site-specific CS-DCT in **c** style features *s*_*j*_ are obtained by encoding a reference image *x*_*j*_. **e** The MURD framework. MURD implements CS-DCT by employing a site-shared content encoder, a site-specific style encoder, a site-shared generator, a site-specific style generator, and a site-specific discriminator for content encoding, style encoding, decoding, style generation, and adversarial learning, respectively. MURD is constrained by five types of losses: consistency losses ${{{{{{{{\mathcal{L}}}}}}}}}_{{{{{{{{\rm{cyc}}}}}}}}}$, ${{{{{{{{\mathcal{L}}}}}}}}}_{{{{{{{{\rm{cont}}}}}}}}}$ and ${{{{{{{{\mathcal{L}}}}}}}}}_{{{{{{{{\rm{sty}}}}}}}}}$, adversarial loss ${{{{{{{{\mathcal{L}}}}}}}}}_{{{{{{{{\rm{adv}}}}}}}}}$, content alignment loss ${{{{{{{{\mathcal{L}}}}}}}}}_{{{{{{{{\rm{ca}}}}}}}}}$, style diversity loss ${{{{{{{{\mathcal{L}}}}}}}}}_{{{{{{{{\rm{sd}}}}}}}}}$, and identity loss ${{{{{{{{\mathcal{L}}}}}}}}}_{{{{{{{{\rm{id}}}}}}}}}$.

Here, we draw inspiration from recent advancements in multi-domain image-to-image translation^23–25^ and introduce a unified framework for simultaneous multi-site harmonization using only a single deep learning model. Our method, called multi-site unsupervised representation disentangler (MURD, Fig. 1), decomposes an image into anatomical content that is invariant across sites and appearance style (e.g., intensity and contrast) that is dependent on each site (Fig. 1a). Harmonized images are generated by combining the content of an image with styles specific to the different sites (Fig. 1b). More specifically, encoding an image in site-invariant and site-specific representations is achieved via two encoders, i.e., a content encoder that captures anatomical structures common across sites and a style encoder that captures style information specific to each site. A site-harmonized image is generated via a generator that combines the extracted content with the style associated with a target site. The target style can be specified by a reference image from a site or by a randomized style code generated by a style generator specific to each site (Fig. 1c, d). The latter allows multiple appearances to be generated in relation to natural style variations associated with each site. MURD is trained with losses that are designed to promote full representation disentanglement and to maximally preserve structural details (Fig. 1e).

## Results

### Multi-site MRI datasets

**Fig. 2:**
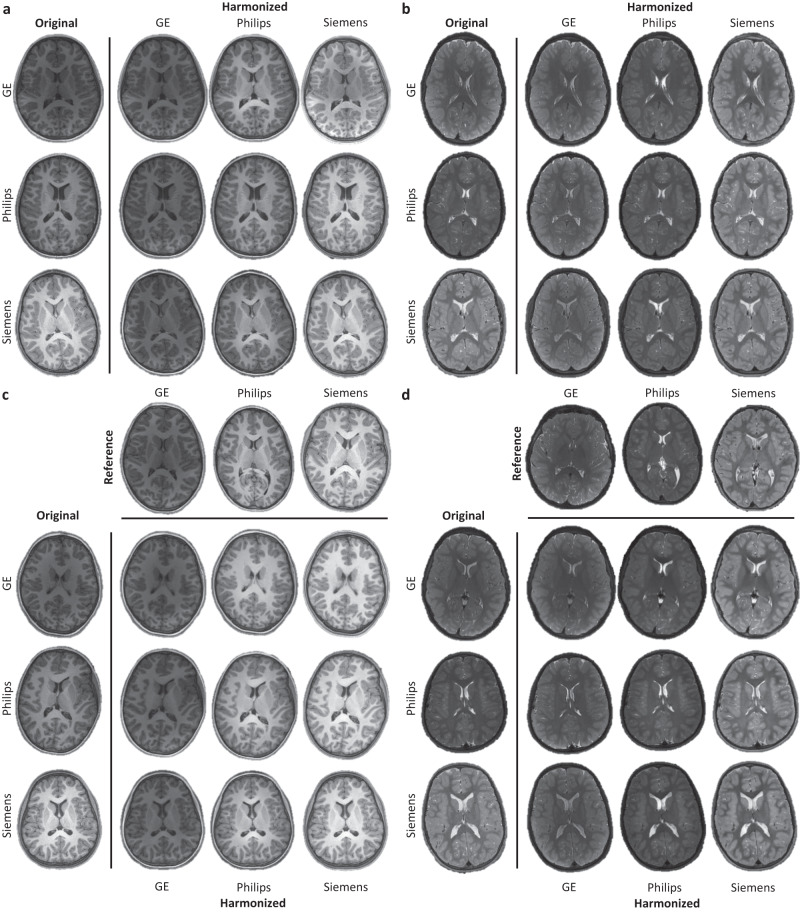
Harmonization of T1- and T2-weighted Images. Site-specific harmonization of **a** T1-weighted images and **b** T2-weighted images. Reference-specific harmonization of **c** T1-weighted images and **d** T2-weighted images. The original images are shown in the first column and the harmonized images are shown for General Electric (GE), Philips, and Siemens respectively, in the second to fourth columns.

We demonstrate the effectiveness of MURD on brain T1- and T2-weighted images of children 9–10 years of age acquired through the ABCD study^1^ using scanners from different vendors, including General Electric (GE), Philips, and Siemens. Informed consent was obtained from all participants^1^. Although the imaging protocols were matched across scanners at the different imaging sites^26^, inter-scanner variability in appearance is still notable (Fig. 2). For simplicity, we group the images and treat them as coming from three *virtual* sites—the GE, Philips, and Siemens sites.

We trained and tested MURD separately for T1- and T2-weighted image volumes. For each modality, we trained MURD using a modest sample size of 20 volumes per vendor. Three datasets per modality were used for testing different aspects of MURD: (1) *Validation Dataset*—A small but diverse dataset of 10 volumes per vendor, carefully selected to be structurally different from the images in the training dataset. The purpose of this testing dataset is to test the effectiveness of MURD beyond the training dataset. (2) *Generalizability Dataset*—A large dataset of 1000 volumes randomly selected for each vendor. This testing dataset is allowed to be deliberately much larger than the training dataset to test the generalizability of MURD. (3) *Human Phantom Dataset*—A traveling human phantom dataset of 1 subject scanned on both GE and Philips scanners, 5 subjects scanned with both GE and Siemens scanners, and 2 subjects scanned with both Philips and Siemens scanners. This testing dataset allows numerical evaluation to ensure that anatomical structures are preserved after harmonization. T1- and T2-weighted images for each subject were aligned using Advanced Normalization Tools (ANTS)^27^.

Note that the training dataset and the three testing datasets are mutually exclusive, with no overlapping samples. Taking into account both modalities and all three vendors, >6000 image volumes were used for evaluation, with 60 central axial slices extracted from each image volume for training and testing. Three adjacent slices were merged into a 2.5D slice.

### MURD harmonizes contrasts and preserves details

We demonstrate the effectiveness of MURD harmonization on T1- and T2-weighted images (Fig. 2). Note that MURD allows harmonization with respect to either a site or a reference image. The former amounts to harmonization with respect to an image randomly drawn from a site image distribution. MURD is remarkably effective in adapting contrast and preserving details when harmonizing images from a source site with a target site (Fig. 2a, b). When the source and target sites are identical, MURD retains both contrast and details. When given a reference image, MURD harmonizes the contrasts of images from a source site with the reference image but preserves the anatomical details of the original images (Fig. 2c, d).

### MURD outperforms state-of-the-art methods

To further demonstrate the effectiveness and superiority of MURD, we compared it with two closely related state-of-the-art unsupervised methods, i.e., DRIT++^28^ and StarGAN-v2^25^, which are designed respectively for dual-domain and multi-domain image-to-image translation. DRIT++ and StarGAN-v2 were implemented and trained as described in the original papers^25,28^. DRIT++ was trained once for every pair of sites. StarGAN-v2 was trained concurrently for multiple sites, similar to MURD. We quantitatively compared the visual quality of the harmonized images using two metrics, i.e., the Frechét inception distance (FID)^29^ and the kernel inception distance (KID)^30^, which reflect distribution discrepancy between two sets of images in a manner that correlates well with human visual judgment^31^. FID and KID are respectively computed based on the Frechét distance and maximum mean discrepancy (MMD) of features from the last average pooling layer of Inception-V3^32^ trained on ImageNet^33^. FID and KID were computed at the slice level for the harmonized images with respect to the training images of the target sites. For site-specific harmonization, 10 target-site harmonized images were generated for each testing image of the source site with 10 randomly generated style codes. For reference-specific harmonization, each image in the source site was harmonized with respect to 10 reference images randomly selected from the testing images of a target site. The results (Supplementary Fig. 1a, b) indicate that MURD outperforms DRIT++ and StarGAN-v2 both in terms of FID and KID. For comparison, reference FID and KID values were computed between the training and testing images from the same site (denoted as “Reference”). The FID and KID values given by MURD are remarkably closer to the reference values than DRIT++ and StarGAN-v2. MURD harmonization of the generalizability dataset (Supplementary Fig. 1c, d) yields FID and KID values that are highly consistent with the validation dataset and close to the reference values. This indicates that, although trained using a modest dataset, the model is generalizable to a much larger dataset.

### MURD efficacy validated via traveling human phantom data

**Fig. 3:**
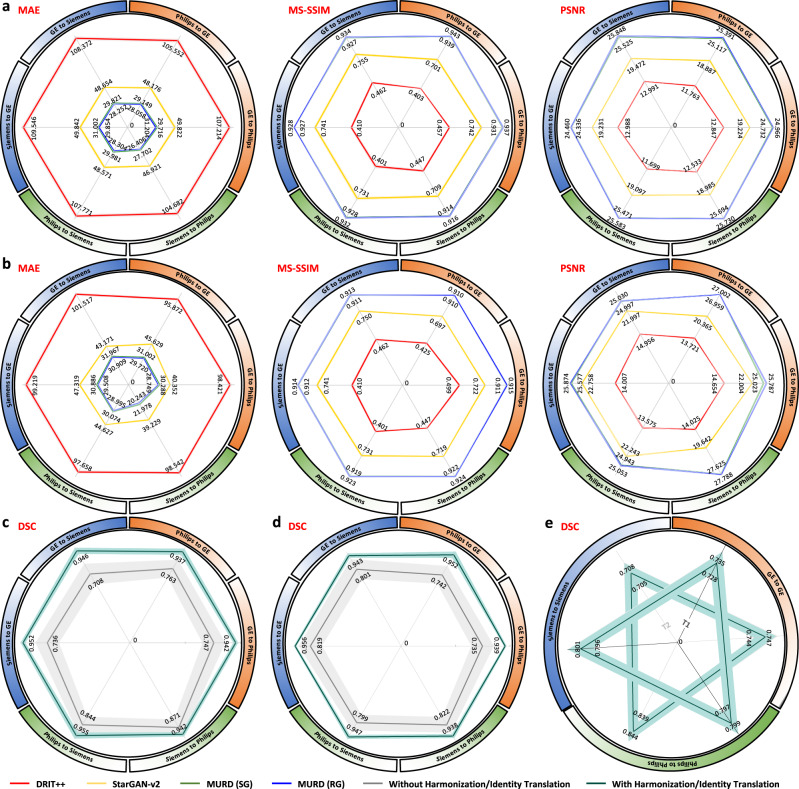
Validation via the traveling human phantom dataset. Evaluation of harmonized, **a** T1-weighted images and, **b** T2-weighted images from the traveling human phantom dataset based on mean absolute error (MAE), multi-scale structural similarity (MS-SSIM), and peak signal-to-noise ratio (PSNR) for the following harmonization tasks: General Electric (GE) to Philips (*n* = 60 slices), Philips to GE (*n* = 60 slices), GE to Siemens (*n* = 600 slices), Siemens to GE (*n* = 600 slices), Philips to Siemens (*n* = 120 slices), and Siemens to Philips (*n* = 120 slices). Segmentation accuracy of, **c** T1-weighted images and, **d** T2-weighted images from the traveling human phantom dataset with and without harmonization based on Dice similarity coefficient (DSC): GE to Philips (*n* = 1 volumes), Philips to GE (*n* = 1 volumes), GE to Siemens (*n* = 5 volumes), Siemens to GE (*n* = 5 volumes), Philips to Siemens (*n* = 2 volumes), and Siemens to Philips (*n* = 2 volumes). **e** Segementation consistency of T1- and T2-weighted images with and without identity translation based on DSC: GE to GE (*n* = 6 volumes), Philips to Philips (*n* = 3 volumes), and Siemens to Siemens (*n* = 7 volumes). The lines show the means, and the shaded regions show the standard errors of the means. MURD yields the best performance in terms of MAE, MS-SSIM, and PSNR. With MURD, segmentation accuracy is improved with harmonization and preserved with identity translation.

The human phantom dataset allows direct quantitative evaluation of the effects of harmonization on consistency of structure and appearance. Based on multiple metrics, including mean absolute error (MAE), multi-scale structural similarity index (MS-SSIM), and peak signal-to-noise ratio (PSNR), MURD substantially outperforms DRIT++ and StarGAN-v2, indicating better harmonization of contrast and preservation of anatomical details (Fig. 3a, b).

### MURD improves tissue segmentation consistency

Segmentation of brain tissues is sensitive to variation in image contrast, and under- or over-segmentation might happen owing to differences in acquisition protocols. We applied Brain Extraction Tool (BET)^34^ and FMRIB’s Automated Segmentation Tool (FAST)^35^ on T1- and T2-weighted images in the human phantom dataset for brain extraction and tissue segmentation. Tissue segmentation consistency before and after harmonization was measured using the Dice similarity coefficient (DSC) with the tissue segmentation maps from the target site as references. The results (Fig. 3c, d) indicate that DSCs are improved remarkably by harmonization using MURD. The tissue segmentation results before and after identity translation (Fig. 3e) demonstrates that DSCs are highly consistent by identity translation using MURD.

### MURD harmonizes volumetric measurements and preserves gender differences

**Fig. 4:**
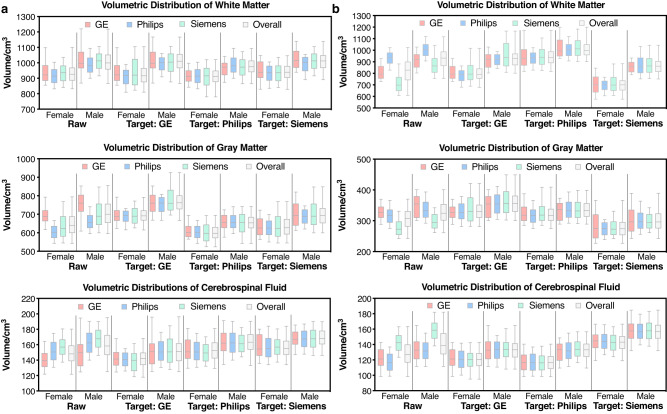
Effects of harmonization on tissue volume distributions. Volumetric distributions of white matter (WM), gray matter (GM), and cerebrospinal fluid (CSF) of unharmonized and harmonized, **a** T1-weighted images and, **b** T2-weighted images from the generalizability dataset. The evaluation was performed based on 500 T1-/T2-weighted images for each gender and each site. The box-and-whisker plots show the medians, lower quartiles, upper quartiles, minima, and maxima.

We demonstrate that MURD reduces inter-site differences in tissue volumetric measurements of gray matter (GM), white matter (WM), and cerebrospinal fluid (CSF) while retaining biological differences due to gender. Brain extraction and tissue segmentation were performed using the T1- and T2-weighted images of each site in the generalizability dataset. Distributions of WM, GM, CSF volumetric measurements were then compared before and after harmonization with respect to the different target sites (Fig. 4a, b). Before harmonization, the distributions differ across sites due to site-specific differences in image appearance, which affect subsequent image processing and hence volumetric measurements. Inter-site differences are removed after harmonization, irrespective of which site is used as the target site (Supplementary Fig. 2a, b). Note that when images from a site are harmonized to the same site, the volumetric distribution is retained. Gender differences captured in the original data, i.e., greater volumes in males, are preserved after harmonization (Supplementary Fig. 2c, d).

### MURD harmonizes across resolutions

**Fig. 5:**
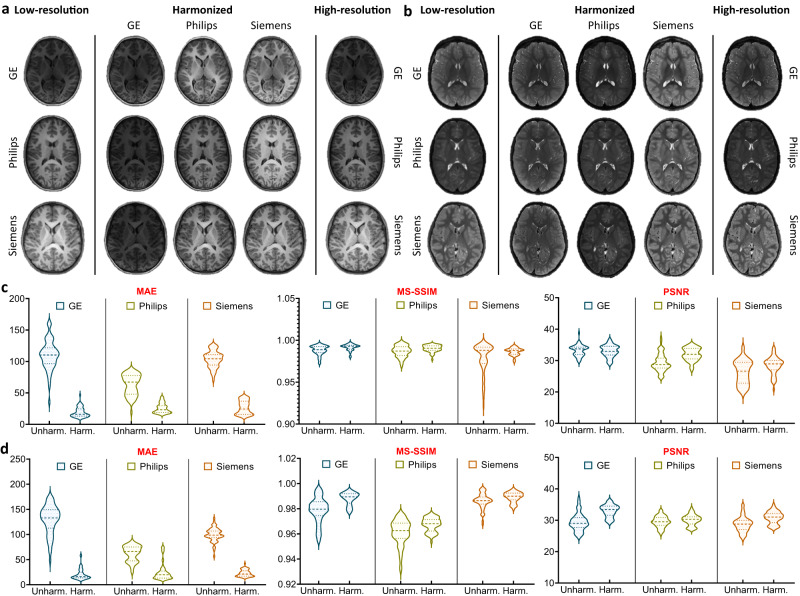
Harmonization Across Resolutions. **a** T1-weighted images and, **b** T2-weighted images of a lower 1.25 mm isotropic resolution harmonized to a higher 1 mm isotropic resolution using reference-specific MURD. Quantitative evaluation conducted for the General Electric (GE), Philips, and Siemens sites with mean absolute error (MAE), multi-scale structural similarity (MS-SSIM), and peak signal-to-noise ratio (PSNR) for, **c** T1-weighted images (*n* = 60,000 slices per site) and, **d** T2-weighted images (*n* = 60,000 slices per site) from the generalizability dataset. (Unharm.: Unharmonized. Harm.: Harmonized).

It is common that multi-site images differ not only in contrast but also in resolution. MURD recovers image details when harmonizing lower-resolution images with respect to higher-resolution images. To demonstrate this, we reduced the effective resolution of the images from 1 mm to 1.25 mm and then recover image details by harmonizing them with respect to images at the original 1 mm resolution. Figure 5a, b show example T1- and T2-weighted images of 1.25 mm resolution harmonized to 1 mm resolution with MURD and Fig. 5c, d present the quantitative results of cross-resolution harmonization for T1- and T2-weighted images from the generalizability dataset, demonstrating high-fidelity detail recovery.

### MURD supports continuous harmonization

**Fig. 6:**
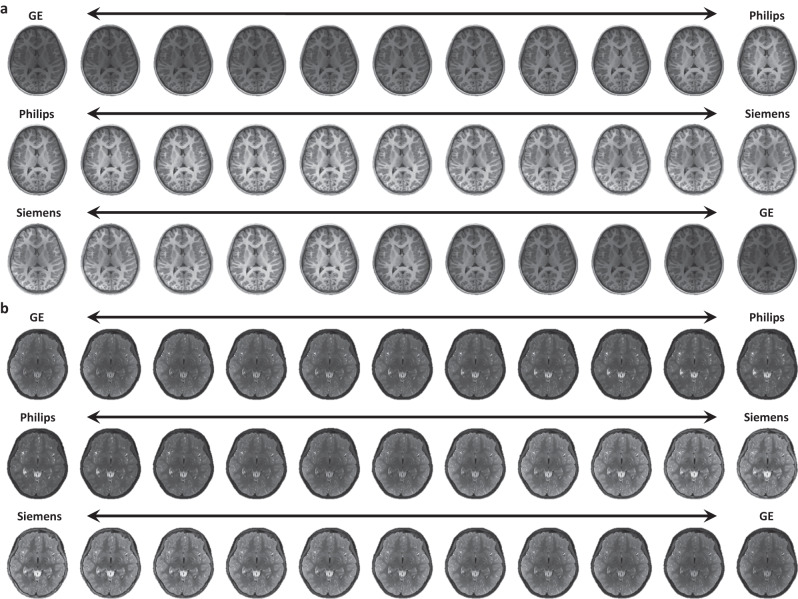
Continuous harmonization. Continuous harmonization of **a** T1-weighted images, and **b** T2-weighted images between two sites, demonstrating that MURD smoothly changes image contrasts without altering anatomical details thanks to its ability to disentangle content and style information.

We further verify the capability of MURD in complete disentanglement of content and style information of MURD via continuous harmonization. We generated images with between-site appearances to aid visual inspection of how appearance changes gradually between sites and whether unwanted anatomical alterations are introduced in the process. Intermediate style features are calculated based on the style features of Site A, *s*_A_, and Site B, *s*_B_, via (1−*β*)*s*_A_ + *βs*_B_, where *β* ∈ [0, 1]. The intermediate style features and the content features of an image are then used to generate an intermediate image. MURD gradually and smoothly changes image appearance without altering anatomical details (Fig. 6a, b).

## Discussion

We have introduced MURD, a unified framework for the simultaneous harmonization of multi-site MR images via unsupervised deep learning. MURD addresses the challenges in MRI harmonization by representation disentanglement learning for the complete decomposition of anatomical content information and appearance style information to ensure only appearance information is altered while generating site-harmonized MR images. We have demonstrated that MURD is remarkably effective in harmonizing MR images by removing non-biological site differences and, at the same time, preserving anatomical details. MURD network training involves only site-labelled images and requires no traveling human phantom data. This flexibility allows data from existing large-scale studies to be harmonized retrospectively without requiring additional data to be collected. Unlike most unsupervised learning-based harmonization methods that require multiple training of models for each two sites, MURD only needs individual training for simultaneously all-site harmonization. In addition, MURD can be incrementally adapted for new sites via knowledge distillation^36^, which only requires optimizing a small number of parameters instead of model training from scratch. This is a substantial advantage in practice since only a small amount of training data is needed from the new sites.

We have shown that MURD yields superior performance over DRIT++^28^ and StarGAN-v2^25^. For every pair of sites, DRIT++ embeds images into a site-invariant content representation capturing information shared across sites and a site-specific style representation. The encoded content features extracted from an image of one site are combined with style features from another site to synthesize the corresponding harmonized image. Learning is unsupervised, and hence paired data is not required. However, DRIT++ is not scalable due to the need to learn all mappings for all site pairs. DRIT++ is also ineffective because it cannot fully utilize the entire training data and can only learn from two sites at each time, causing it to miss global features that can be learned from images of all sites. Failure to fully utilize training data likely limits the quality of generated images. Unlike DRIT++, StarGAN-v2 is scalable and performs image-to-image translations for multiple sites using only a single model. It has been applied to the problem of MRI harmonization^37,38^ with promising results. In addition to greater scalability, StarGAN-v2 generates images of higher visual quality owing to its ability to jointly consider the information offered by images from all sites. StarGAN-v2, however, does not explicitly disentangle images into structural and appearance information. This introduces the possibility of altering anatomical details during harmonization via style transfer. In contrast, MURD enforces explicit disentanglement of content and style features by jointly considering images from all sites, allowing it to produce harmonized images with diverse appearances and greater details (Supplementary Note 1 and Supplementary Figs. 4–9). Disentanglement safeguards harmonization against altering image anatomical contents (Supplementary Note 2 and Supplementary Fig. 10) and allows gradual and controllable harmonization via interpolation of style features.

The harmonization target is specified for DRIT++ and StarGAN-v2 by a reference image. The appearance of the harmonized image is determined by the style features extracted from the reference image. In addition to a reference image, MURD offers the option of specifying the harmonization target by a site label, which determines the output branch of the style generator and the style encoder. A latent code sampled from the standard Gaussian distribution determines an appearance specific to the site.

A recent MRI harmonization method, called CALAMITI^39^ rely on intra-site supervised image-to-image translation and unsupervised domain adaptation for multi-site harmonization. This requires training a disentangled representation model with intra-site multi-contrast images (T1- and T2-weighted images) of the same subjects and retraining the model for a new site via domain adaptation^40^. Unlike CALAMITI, MURD requires only images from a single contrast and can learn multi-site harmonization simultaneously without needing fine-tuning or retraining. In addition, MURD utilizes a 2.5D training strategy, unlike the 2D strategy used in CALAMITI. Essentially, each 2.5D slice integrates a slice and its two adjacent slices as a three-channel image, allowing MURD to use information in the third dimension to generate spatially smooth 3D volumes without abrupt anatomical changes. This strategy also reduces memory requirements by avoiding the need for training a 3D fusion network as in CALAMITI.

MURD is currently evaluated using only MR images of healthy individuals. Atypical appearances resulting, for example, from brain tumors may interfere with harmonization. Fortunately, the unsupervised learning nature of MURD implies that images with abnormal appearances can be easily incorporated into the model training process. Appearance changes associated with abnormal conditions can be learned as part of the content information in MURD to ensure that important diagnostic information is preserved during harmonization.

In summary, MURD is a highly-effective and unified framework for multi-site harmonization of MR images, not only for generating harmonized images from different sites simultaneously using a single model but also for achieving remarkable performance without human traveling phantoms. The capability of complete disentanglement of anatomical content and appearance style representations enables generating harmonized images by the combination of site-invariant content features and site-specific style features. Results on more than 6,000 multi-site T1- and T2-weighted images demonstrate the remarkable performance of MURD in realistic MRI harmonization and great potential to large-scale multi-site studies.

## Methods

We consider the multi-site harmonization problem as image-to-image translation among multiple sites and propose an end-to-end multi-site unsupervised representation disentangler (MURD, Fig. 1) to learn content-style disentangled cycle translation mappings that translate images forward and backward between any two sites. We employ two encoders to respectively embed each image in a site-invariant content representation, which captures anatomical information, and a site-specific style representation, which captures appearance information, and a generator to produce a harmonized image using the encoded content features and site-specific style features. MURD extends a recent representation disentanglement learning framework, called DUNCAN^41^, for efficient multi-site image translation.

### Content-style disentangled cycle translation (CS-DCT)

Given image sets ${\{{{{{{{{{\mathcal{X}}}}}}}}}_{i}\}}_{i = 1}^{N}$ from *N* distinct sites, MURD utilizes content-style disentangled cycle translation (CS-DCT) to disentangle each image x_*_ (* = *i*, *j*; *i* ≠ *j*) into a site-invariant content representation c_*_ and a site-specific style representation s_*_, and the disentangled representations can in turn be combined to generate reconstructed image ${\bar{x}}_{* }$ of x_*_ and harmonized image ${\tilde{x}}_{\star }$ (⋆ = *j*, *i*) of x_*_ (Fig. 1a and b). CS-DCT, realized with sequential forward and backward translation, can be site-specific (Fig. 1c) or reference-specific (Fig. 1d). MURD jointly learns site-invariant content features *c*_*i*_ and site-specific style features *s*_*i*_ from image *x*_*i*_, and utilize generator to construct the harmonized image ${\tilde{x}}_{j}\in {{{{{{{{\mathcal{X}}}}}}}}}_{j}$ using content features *c*_*i*_ and style features *s*_*j*_ in forward translation. Style features *s*_*j*_ are generated by a style generator or extracted from a reference image ${x}_{j}\in {{{{{{{{\mathcal{X}}}}}}}}}_{j}$. In backward translation, MURD extracts the content features ${\tilde{c}}_{j}$ from the harmonized image ${\tilde{x}}_{j}$, which are in turn fed with style features *s*_*i*_ to generator *G* to reconstruct image ${\hat{x}}_{i}$, which is required to be consistent with the input image *x*_*i*_.

### The MURD achitecture

MURD implements CS-DCT in a end-to-end manner via five modules: (i) A content encoder *E*^C^ (Supplementary Fig. 3a), shared for all sites, to extract content features c_*_ (* = *i*, *j*) from an image x_*_; (ii) A style encoder ${E}_{i}^{{{{{{{{\rm{S}}}}}}}}}$ (Supplementary Fig. 3b) for each site *i* to extract site-specific style features *s*_*i*_ from an image *x*_*i*_; (iii) A generator *G* (Supplementary Fig. 3c), shared for all sites, to synthesize images using content and style features; (iv) A style generator ${G}_{j}^{{{{{{{{\rm{S}}}}}}}}}$ (Supplementary Fig. 3d) for each site *j* to yield style features *s*_*j*_ that reflect the appearance style of images from the site; and (v) A discriminator *D*_*j*_ (Supplementary Fig. 3e) for each site *j* to distinguish between real and generated images. See Supplementary Methods for details on network architectures.

Specifically, as illustrated in Fig. 1e, given an input image *x*_*i*_ from image set ${{{{{{{{\mathcal{X}}}}}}}}}_{i}$, content encoder *E*^C^ and style encoder ${E}_{i}^{{{{{{{{\rm{S}}}}}}}}}$, respectively, extract content features *c*_*i*_ and style features *s*_*i*_. For a site *j* ≠ *i*, style generator ${G}_{j}^{{{{{{{{\rm{S}}}}}}}}}$ takes a latent code *z* randomly sampled from a standard normal distribution ${{{{{{{\mathcal{N}}}}}}}}(0,1)$ as input to create a *j*-site style features *s*_*j*_. With content features *c*_*i*_ and style features *s*_*j*_, generator *G* constructs harmonized image ${\tilde{x}}_{j}$, i.e., ${\tilde{x}}_{j}=G({c}_{i},{s}_{j})$, which the discriminator *D*_*j*_ then classifies as being either real or fake using an adversarial loss ${{{{{{{{\mathcal{L}}}}}}}}}_{{{{{{{{\rm{adv}}}}}}}}}={\mathbb{E}}[\log {D}_{i}({x}_{i})]+{\mathbb{E}}[\log (1-{D}_{j}({\tilde{x}}_{j}))]$. Content encoder *E*^C^ and style encoder ${E}_{j}^{{{{{{{{\rm{S}}}}}}}}}$ are used to extract content features ${\tilde{c}}_{j}$ and style features ${\tilde{s}}_{j}$ from ${\tilde{x}}_{j}$. The consistency between *c*_*i*_ and ${\tilde{c}}_{j}$ is enforced by a pixel-wise content consistency loss ${{{{{{{{\mathcal{L}}}}}}}}}_{{{{{{{{\rm{cont}}}}}}}}}=\,\parallel {E}^{{{{{{{{\rm{C}}}}}}}}}({x}_{i})-{E}^{{{{{{{{\rm{C}}}}}}}}}({\tilde{x}}_{j}){\parallel }_{1}=\,\parallel {c}_{i}-{\tilde{c}}_{j}{\parallel }_{1}.$ The consistency between *s*_*j*_ and ${\tilde{s}}_{j}$ is enforced by a pixel-wise style consistency loss ${{{{{{{{\mathcal{L}}}}}}}}}_{{{{{{{{\rm{sty}}}}}}}}}=\,\parallel {E}_{i}^{{{{{{{{\rm{S}}}}}}}}}({x}_{i})-{E}_{j}^{{{{{{{{\rm{S}}}}}}}}}({\tilde{x}}_{j}){\parallel }_{1}=\,\parallel {s}_{i}-{\tilde{s}}_{j}{\parallel }_{1}$. Content site-invariance is enforced by a content alignment loss ${{{{{{{{\mathcal{L}}}}}}}}}_{{{{{{{{\rm{ca}}}}}}}}}={{{{{{{\rm{KL}}}}}}}}({{{{{{{\mathcal{N}}}}}}}}({c}_{i},I)\parallel {{{{{{{\mathcal{N}}}}}}}}(0,I))$, where KL( ⋅ ∥ ⋅ ) is the Kullback-Leibler divergence and *I* is an identity matrix. Content features *c*_*i*_ are randomly perturbed during the feed-forward step, i.e., ${c}_{i}={E}^{{{{{{{{\rm{C}}}}}}}}}({x}_{i})+\eta ,\quad \eta \sim {{{{{{{\mathcal{N}}}}}}}}(0,I)$. To allow diverse intra-site styles, the style generator is explicitly regularized with a style diversity loss ${{{{{{{{\mathcal{L}}}}}}}}}_{{{{{{{{\rm{sd}}}}}}}}}$ at image level, i.e., ${{{{{{{{\mathcal{L}}}}}}}}}_{{{{{{{{\rm{sd}}}}}}}}}=\,\parallel G({c}_{i},{s}_{j})-G({c}_{i},{s}_{j}^{{\prime} }){\parallel }_{1}=\,\parallel {\tilde{x}}_{j}-{\tilde{x}}_{j}^{{\prime} }{\parallel }_{1}$, where if *z* is the latent code associated with *s*_*j*_, ${s}_{j}^{{\prime} }$ is generated from a latent code ${z}^{{\prime} }\ne z$ randomly sampled from a standard normal distribution ${{{{{{{\mathcal{N}}}}}}}}(0,1)$. When the content and style features are completely disentangled, the style feature *s*_*j*_ is consistent with style feature ${\tilde{s}}_{j}$. This consistency is constrained by a style consistency loss ${{{{{{{{\mathcal{L}}}}}}}}}_{{{{{{{{\rm{sty}}}}}}}}}$ at the pixel level, i.e., ${{{{{{{{\mathcal{L}}}}}}}}}_{{{{{{{{\rm{sty}}}}}}}}}=\,\parallel {s}_{i}-{\tilde{s}}_{j}{\parallel }_{1}$. The reconstructed image ${\hat{x}}_{i}$ of *x*_*i*_ is produced by generator *G* using content features ${\tilde{c}}_{j}$ and style features *s*_*i*_, i.e., ${\hat{x}}_{i}=G({\tilde{c}}_{j},{s}_{i})$. The consistency between *x*_*i*_ and ${\hat{x}}_{i}$ is ensured by a pixel-wise and gradient-wise cycle consistency loss ${{{{{{{{\mathcal{L}}}}}}}}}_{{{{{{{{\rm{cyc}}}}}}}}}$, i.e., ${{{{{{{{\mathcal{L}}}}}}}}}_{{{{{{{{\rm{cyc}}}}}}}}}=\,\parallel {x}_{i}-{\hat{x}}_{i}{\parallel }_{1}+{\lambda }_{{{{{{{{\rm{g}}}}}}}}}\parallel g({x}_{i})-g({\hat{x}}_{i}){\parallel }_{1}$, where *g*( ⋅ ) is the image gradient function and *λ*_g_ is the loss weight for the gradient loss term. Furthermore, an identity image ${\bar{x}}_{i}$ can also be constructed by generator *G* using content features *c*_*i*_ and style features *s*_*i*_, which are identical to *x*_*i*_ when *c*_*i*_ and *s*_*i*_ are completely disentangled. We devise an identity loss to measure the pixel-wise difference between *x*_*i*_ and ${\bar{x}}_{i}$ as ${{{{{{{{\mathcal{L}}}}}}}}}_{{{{{{{{\rm{id}}}}}}}}}=\,\parallel {x}_{i}-G({c}_{i},{s}_{i}){\parallel }_{1}=\parallel {x}_{i}-{\bar{x}}_{i}{\parallel }_{1}$. All modules are optimized with total loss function ${{{{{{{\mathcal{L}}}}}}}}={{{{{{{{\mathcal{L}}}}}}}}}_{{{{{{{{\rm{adv}}}}}}}}}+{\lambda }_{{{{{{{{\rm{cont}}}}}}}}}{{{{{{{{\mathcal{L}}}}}}}}}_{{{{{{{{\rm{cont}}}}}}}}}+{\lambda }_{{{{{{{{\rm{ca}}}}}}}}}{{{{{{{{\mathcal{L}}}}}}}}}_{{{{{{{{\rm{ca}}}}}}}}}+{\lambda }_{{{{{{{{\rm{sd}}}}}}}}}{{{{{{{{\mathcal{L}}}}}}}}}_{{{{{{{{\rm{sd}}}}}}}}}+{\lambda }_{{{{{{{{\rm{sty}}}}}}}}}{{{{{{{{\mathcal{L}}}}}}}}}_{{{{{{{{\rm{sty}}}}}}}}}+{\lambda }_{{{{{{{{\rm{cyc}}}}}}}}}{{{{{{{{\mathcal{L}}}}}}}}}_{{{{{{{{\rm{cyc}}}}}}}}}+{\lambda }_{{{{{{{{\rm{id}}}}}}}}}{{{{{{{{\mathcal{L}}}}}}}}}_{{{{{{{{\rm{id}}}}}}}}}$, where *λ*_cont_, *λ*_ca_, *λ*_sd_, *λ*_sty_, *λ*_cyc_, and *λ*_id_ are loss weights used for controlling the contributions of the respective terms in the loss function. Training ends when all modules are optimized, such that the optimized discriminator classifies the harmonized images into one of two categories with equal probability. See Supplementary Note 3 and Supplementary Figs. 11–17 for the effects of the individual loss functions.

During inference, content features *c*_*i*_ of input image *x*_*i*_ are extracted using content encoder *E*^C^, and style features *s*_*j*_ are either generated using style generator ${G}_{j}^{{{{{{{{\rm{S}}}}}}}}}$ or extracted from a reference image *x*_*j*_. Harmonized image ${\tilde{x}}_{j}$ is created by generator *G* using *c*_*i*_ and *s*_*j*_, i.e., ${\tilde{x}}_{j}=G({c}_{i},{s}_{j})$.

### Implementation details

MURD was implemented using Tensorflow. Evaluation was based on a machine with a CPU (i.e., Intel i7-8700K) and a GPU (i.e., NVIDIA GeForce GTX 1080Ti 11GB). The ADAM optimizer with 1 × 10^−4^ learning rate was utilized for minimization based on the loss function. For all datasets, every three adjacent slices in each volume were inserted into three channels. Each channel was then normalized to have a range between −1 and 1 and zero-padded to 256 × 256. The weights of loss functions are core factors that affect the overall performance. We determined these weights based on the following considerations. MURD is primarily designed for pixel-wise and semantic cycle-consistency translation; therefore, the weight of cycle consistency loss *λ*_cyc_ takes a larger value. In addition to cycle consistency, MURD performs pixel-wise identity translation based on the idea of self-reconstruction. This results in a large weight *λ*_id_ for identity loss as for *λ*_cyc_. Another critical component of MURD is content-style representation disentanglement for encoding and entanglement for decoding in each cycle translation. We set the corresponding loss weights *λ*_cont_ and *λ*_sty_ to the same value as *λ*_cyc_. To satisfy the invariance property of content features during harmonization (i.e., all content features should be in a common latent space), we assigned a relatively small weight for the content alignment loss. The style diversity loss designed for style generator is used to capture the intra-site diversity of generated style features. The weight for this loss is set to be unit to encourage contrast variability. For all datasets, we thus used *λ*_adv_ = 1, *λ*_cont_ = 10, *λ*_ca_ = 0.01, *λ*_sd_ = 1, *λ*_sty_ = 10, *λ*_cyc_ = 10, *λ*_g_ = 0.1, and *λ*_id_ = 10 for the corresponding losses.

### Supplementary information

Supplementary Materials PDF File

## Data Availability

The ABCD data is available to the scientific community via the National Institute of Mental Health Data Archive (https://nda.nih.gov/).

## Code availability

The source code for multi-site MURD is available at https://harmonization.yaplab.io.
