# Supplementary material for: Learning multi-site harmonization of magnetic resonance images without traveling human phantoms
Source: Commun Eng. Author manuscript; Available in PMC 2025 Jan 5. (PMC10898625; doi:10.1038/s44172-023-00140-w)
Supplement: supplementary [file NIHMS1963956-supplement-supplementary.pdf]

Supplementary Materials for "Learning Multi-Site Harmonization of Magnetic Resonance Images Without Traveling Human Phantoms"

Siyuan Liu, Pew-Thian Yap

Supplementary Methods: Network Architecture

Supplementary Figure 3 shows the architectures of the components of MURD, including the content encoder E_i^{C}, style encoder E_i^{S}, generator G, style generator G_i^{S}, and discriminator D_j, where i and j ($j \neq i$) denote respectively the i-th and j-th sites. The architectures of the components of MURD are illustrated in Supplementary Figure 3(A)-(D) and are described next.

Content Encoder Content encoder E^{C} is shared among all sites and extracts the content features c_i of an input image x_i through three convolutional blocks and 4 residual blocks. Each convolutional block is composed of three sequential layers, i.e., convolution, instance normalization (IN)[1], and leaky ReLU (LReLU) activation. Each residual block consists of a nonlinear mapping branch and a shortcut branch. The nonlinear mapping branch is constructed by a series of layers, i.e., convolution, IN, LReLU, convolution, and IN. The shortcut branch is an identity mapping of the block input. We use an IN layer instead of a batch normalization layer[1] to accelerate model convergence and maintain independence between features. All normalized feature maps are activated by LReLU with a negative slope of 0.2.

Style Encoder Style encoder E_i^{S} is composed of site-shared and site-specific subnetworks. The site-shared subnetwork is constructed by a convolution layer, four pre-activation residual blocks, and a global average pooling layer. The site-specific subnetwork is composed of N fully connected layers corresponding to the N individual sites. The pre-activation residual block is constructed by integrating LReLU activation followed by a convolution layer with unit stride into a residual block, where an average pooling layer is adopted to downsample the intermediate features and the shortcut branch is implemented by an average pooling layer and a convolution layer with unit kernel size and stride. Note that we extract style features without IN layers since IN removes feature means and variances, which contain important style information. The output dimension is set to 64. Style features s_i have the same dimension.

Generator Site-shared generator G merges content features c_i and style features s_j to create a harmonized image $\tilde{x}_j$ using four residual blocks identical to the content encoder, two upsampling blocks and a convolution layer with hyperbolic tangent (tanh) activation. The upsampling block consists of deconvolution, IN, and LReLU activation.

Style Generator Style generator G_j^{S} consists of a multilayer perception (MLP) with N output branches. Six fully connected layers are shared among all sites, followed by a fully connected layer for each site. We set the dimensions of the latent code, the hidden layer, and the style features to 16, 256, and 64, respectively. We randomly sample the latent code z from the standard Gaussian distribution.

Discriminator Discriminator D_j consists of site-shared and site-specific subnetworks, similar to the style encoder. Specifically, three convolutional blocks and a global average pooling are shared among all sites, followed by a specific fully connected layer for real/fake classification for each site.

Supplementary Notes 1: Visual Comparison

Example T1-weighted and T2-weighted images harmonized using various methods with respect to different sites, i.e., General Electric (GE), Philips, and Siemens, and corresponding difference maps are shown in Supplementary Figures 4–9. Site-specific (SS) and reference-specific (RS) harmonization using MURD yields detail-preserved images that are significantly closer to the reference images.

Supplementary Notes 2: Consistency of Content Representation

Example content representations of a T1-weighted GE image and a T1-weighted Philips image of a subject from the traveling human phantom dataset are shown in Supplementary Figure 10. The content features are highly consistent across scanners indicating complete disentanglement from style features via MURD.

Supplementary Notes 3: Ablation Study

To understand the effects of each loss function, we conducted an ablation study using T1- and T2-weighted images from the traveling human phantom dataset. We removed loss functions $\mathcal{L}_{\mathrm{sty}}$, $\mathcal{L}_{\mathrm{cont}}$, $\mathcal{L}_{\mathrm{cyc}}$, and $\mathcal{L}_{\mathrm{ca}}$ independently, and then evaluated the performance of the resulting networks. All training hyper-parameters were matched with those used in MURD. Example harmonized T1- and T2-weighted images and corresponding difference maps are shown in Supplementary Figures 11–16. Numerical results are summarized in Supplementary Figure 17. MURD trained with all losses yields the best performance qualitatively and quantitatively for all evaluation metrics.

Supplementary Table 1: Existing MRI harmonization methods

Category		Method	Summary
Statistics	Intensity normalization	Histogram matching [2--4]	Align distributions of voxel intensity values based on an image template constructed from several control subjects.
		White stripe [5]	Normalize intensity values based on patches of normal appearing white matter (NAWM). Rescaled intensity values are biologically interpretable as units of NAWM.
		Multi-site image harmonization by cumulative distribution function alignment (MICA) [6]	Transform voxel intensity values of one scan to align with the intensity cumulative distribution function (CDF) of a second scan.
	Batch effect adjustment	Removal of Artificial Voxel Effect by Linear regression (RAVEL) [7]	Estimate using a control region of the brain the latent factors of unwanted variation common to all voxels.
		Combating batch effects (ComBat) [8,9]	Identify batch-specific transformation to express all data in a common representation.
Learning	Machine learning	Regression ensembles with patch learning for image contrast agreement (REPLICA) [10]	Supervised training of a random forest for nonlinear regression of a target contrast.
		NeuroHarmony [11]	Supervised training of a random forest for nonlinear regression of a target contrast determined based on prescribed regions.
	Deep learning	DeepHarmony [12]	Supervised training of a U-Net to produce images with a consistent contrast.
		Disentangled Latent Space (DLS) [13]	Supervised training of a deep neural network using disentangled anatomical and contrast components.
		Unlearning of dataset bias [14]	Supervised training of a deep neural network to learn scanner-invariant features and then reducing scanner influence on network predictions in tasks of interest.
		StarGAN-v2 [15,16]	Unsupervised training of a style generator and a GAN jointly using multi-site images.
		CALAMITI [17]	Unsupervised training of a disentangled representation model with intra-site multi-contrast images of the same subjects and retraining the model for a new site via domain adaptation.
		**MURD**	**Unsupervised** harmonization of images using **anatomy-preserving content and style disentanglement learned jointly for multi-sites without relying on multiple contrasts**.

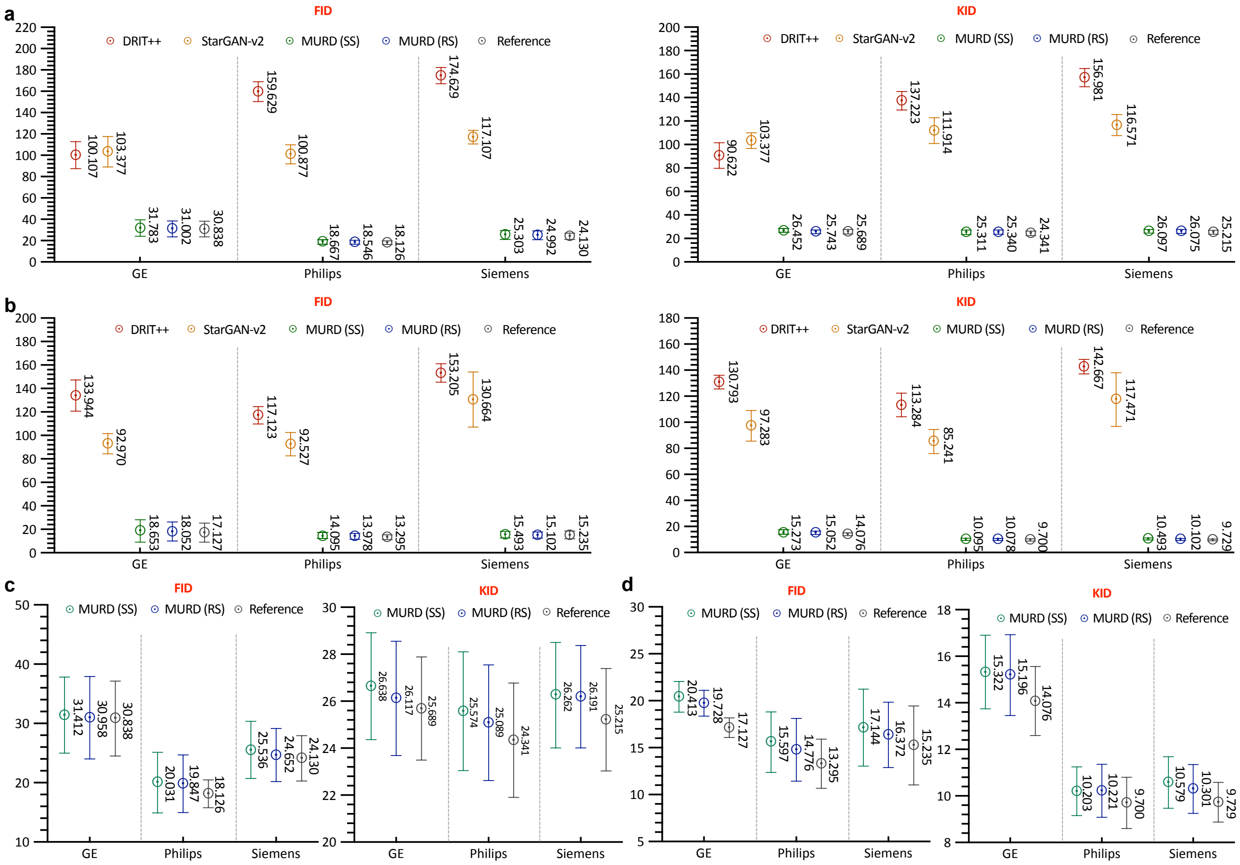

Supplementary Figure 1: Numerical Evaluation of Harmonization Outcomes. Quantitative evaluation conducted for the General Electric (GE), Philips, and Siemens sites using FID and KID as metrics for, **a**, T1-weighted images ($n = 600$ slices per site) and, **b**, T2-weighted images ($n = 600$ slices per site) from the validation dataset, and, **c**, T1-weighted images ($n = 60000$ slices per site) and, **d**, T2-weighted images ($n = 60000$ slices per site) from the generalizability dataset. Bullseyes show the means and error bars show the standard errors on the means. MURD, both site-specific (SS) and reference-specific (RS), yields lower FID and KID values that are closer to the reference values than DRIT++[18] and StarGAN-v2[19]. The FID and KID values for the generalizability dataset are largely consistent with those of the validation dataset.

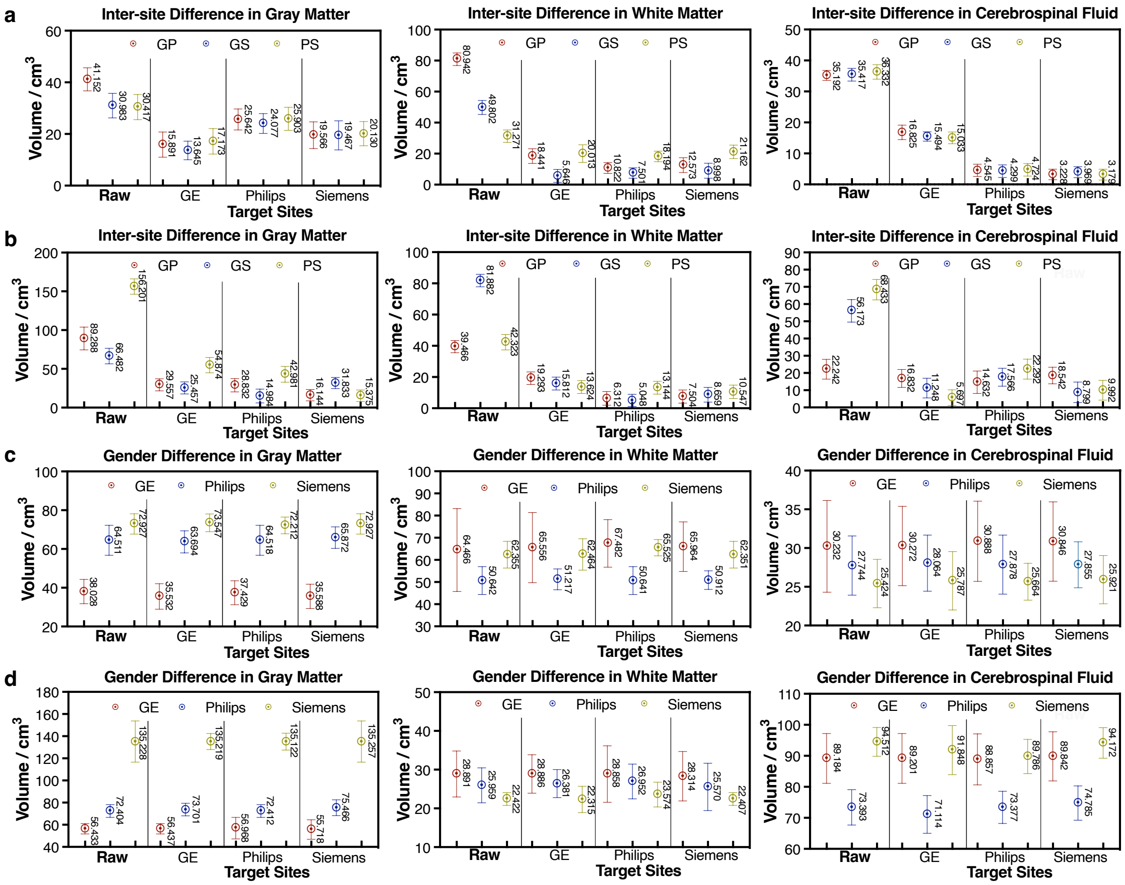

Supplementary Figure 2: Inter-site and Gender Differences in Tissue Volume Distributions. Inter-site and gender difference in volumetric distributions of gray matter (GM), white matter (WM), and cerebrospinal fluid (CSF) of harmonized, **a**, **c**, T1-weighted images and, **b**, **d**, T2-weighted images from the generalizability dataset. Evaluation was performed with 500 T1-/T2-weighted images for each gender and each site. GP, GS, and PS denote respectively inter-site differences between General Electric (GE) and Philips, between GE and Siemens, and between Philips and Siemens. Bullseyes and error bars respectively indicate the absolute differences of means and the standard deviations of differences of means.

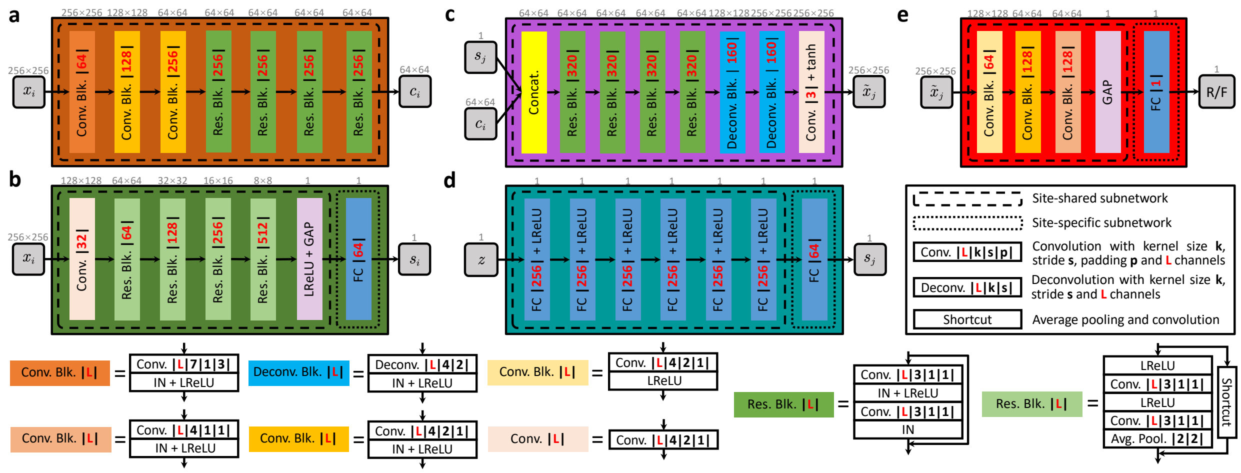

Supplementary Figure 3: Network Architecture. **a**, Network architecture of content encoder E^{C}. **b**, Network architecture of style encoder E_i^{S}. **c**, Network architecture of generator G. **d**, Network architecture of style generator G_j^{S}. **e**, Network architecture of discriminator D_j.

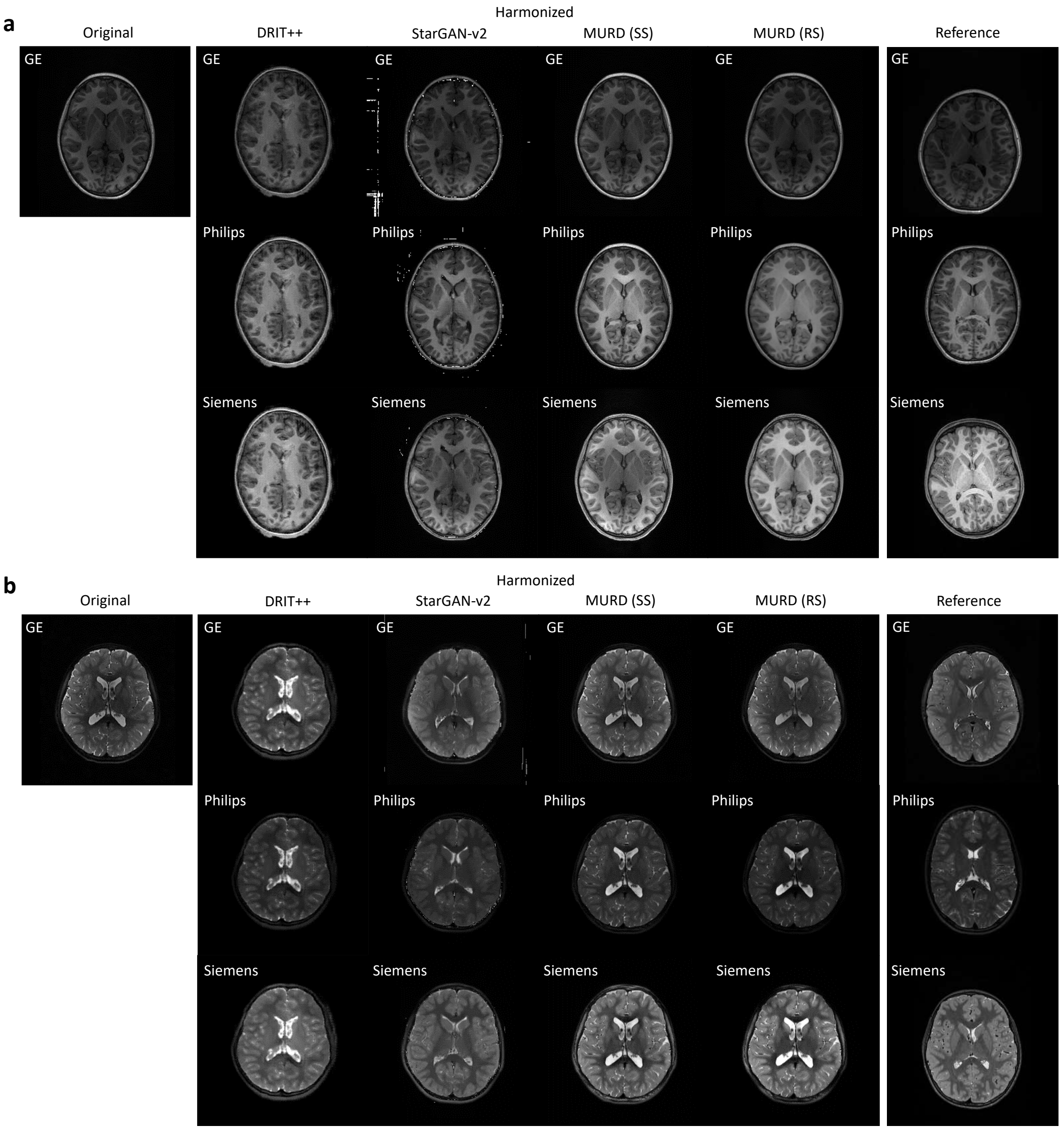

Supplementary Figure 4: Harmonization of General Electric (GE) Images. **a**, T1-weighted and, **b**, T2-weighted images harmonized using various methods with respect to GE, Philips, and Siemens sites. In **a** and **b**, the first column is the original GE image. The second to fifth columns are respectively the harmonized images given by DRIT++, StarGAN-v2, site-specific (SS) MURD and reference-specific (RS) MURD. The last column are reference images from GE, Philips and Siemens sites.

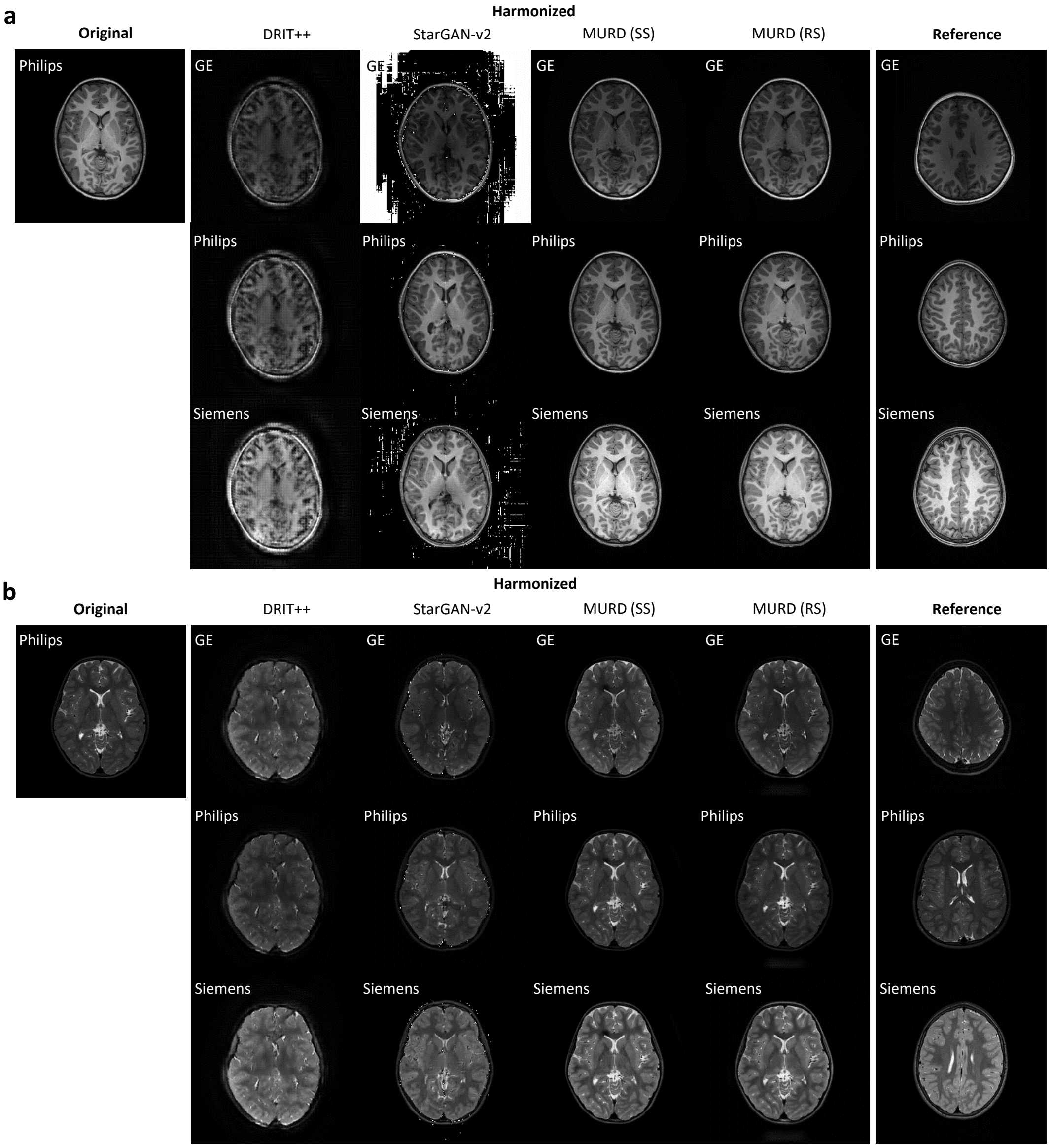

Supplementary Figure 5: Harmonization of Philips Images. **a**, T1-weighted and, **b**, T2-weighted images harmonized using various methods with respect to General Electric (GE), Philips, and Siemens sites. In **a** and **b**, the first column is the original Philips image. The second to fifth columns are respectively the harmonized images given by DRIT++, StarGAN-v2, site-specific (SS) MURD and reference-specific (RS) MURD. The last column are reference images from GE, Philips and Siemens sites.

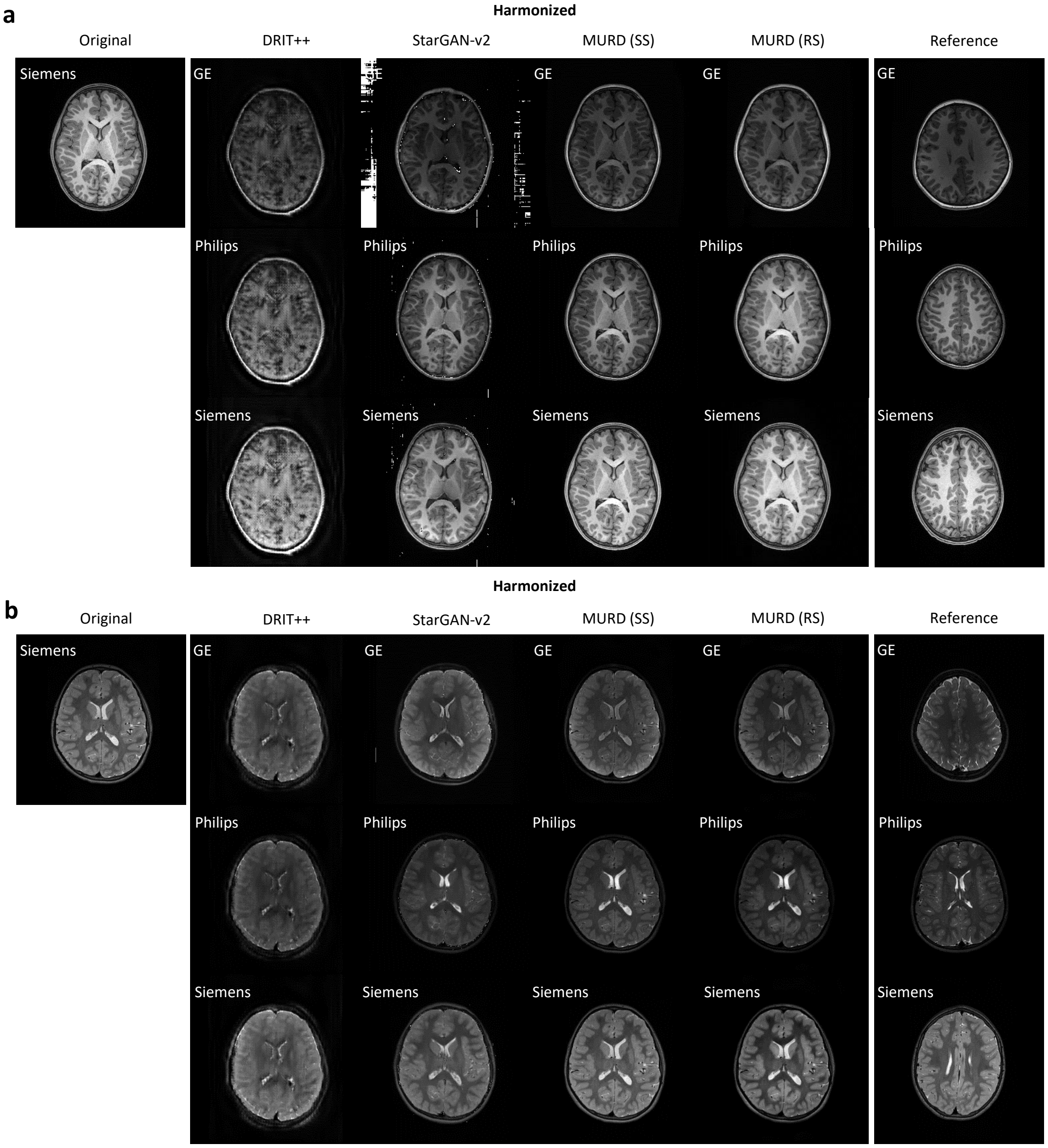

Supplementary Figure 6: Harmonization of Siemens Images. a, T1-weighted and, **b**, T2-weighted images harmonized using various methods with respect to GE, Philips, and Siemens sites. In **a** and **b**, the first column is the original Siemens image. The second to fifth columns are respectively the harmonized images given by DRIT++, StarGAN-v2, site-specific (SS) MURD and reference-specific (RS) MURD. The last column are reference images from GE, Philips and Siemens sites.

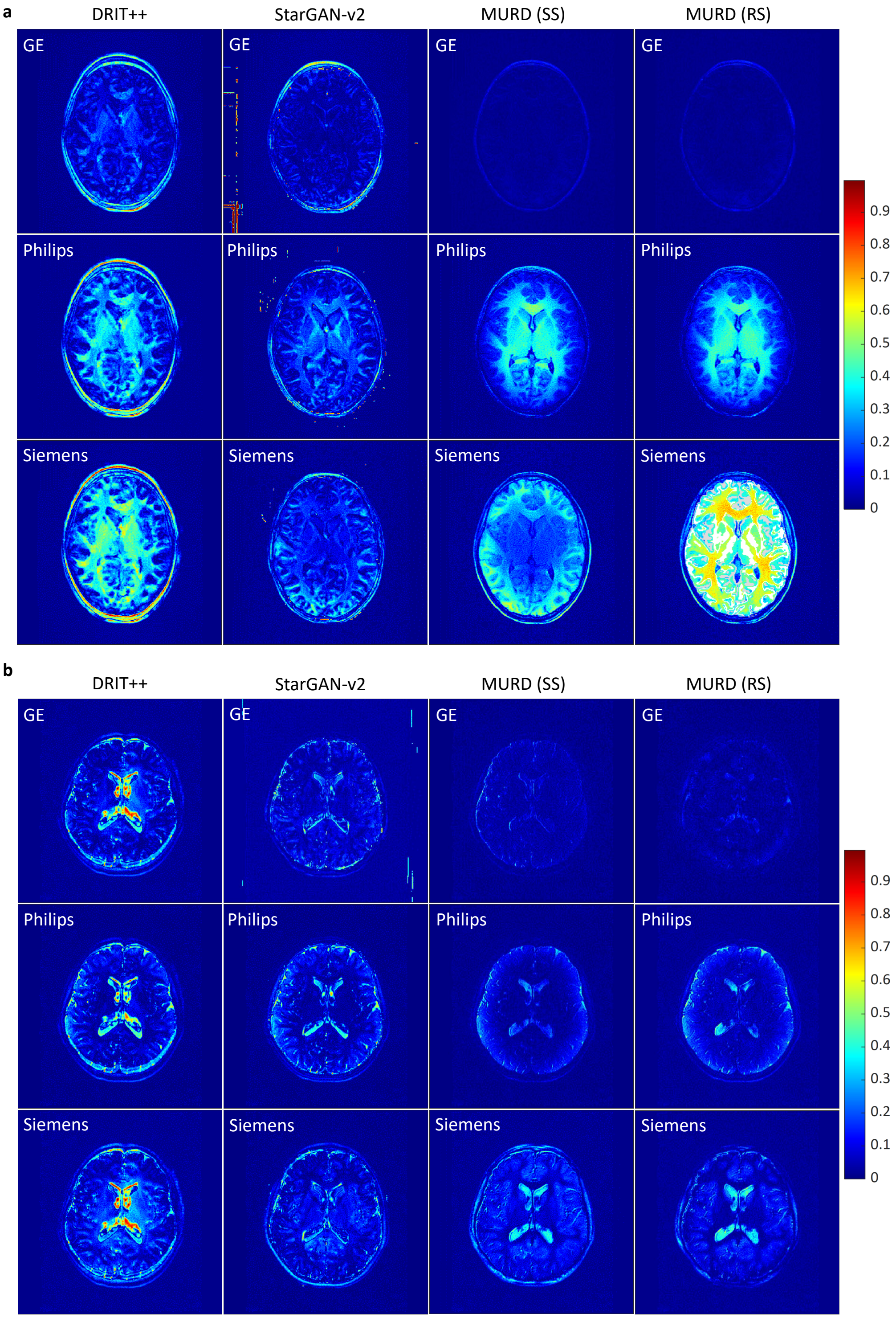

Supplementary Figure 7: Difference maps of Harmonized General Electric (GE) Images and Corresponding Traveling Phanthom Images. Difference maps of, **a**, T1-weighted and, **b**, T2-weighted images harmonized using various methods with respect to GE, Philips, and Siemens sites. In **a** and **b**, the first to fourth columns are respectively difference maps of harmonized images given by DRIT++, StarGAN-v2, site-specific (SS) MURD, and reference-specific (RS) MURD.

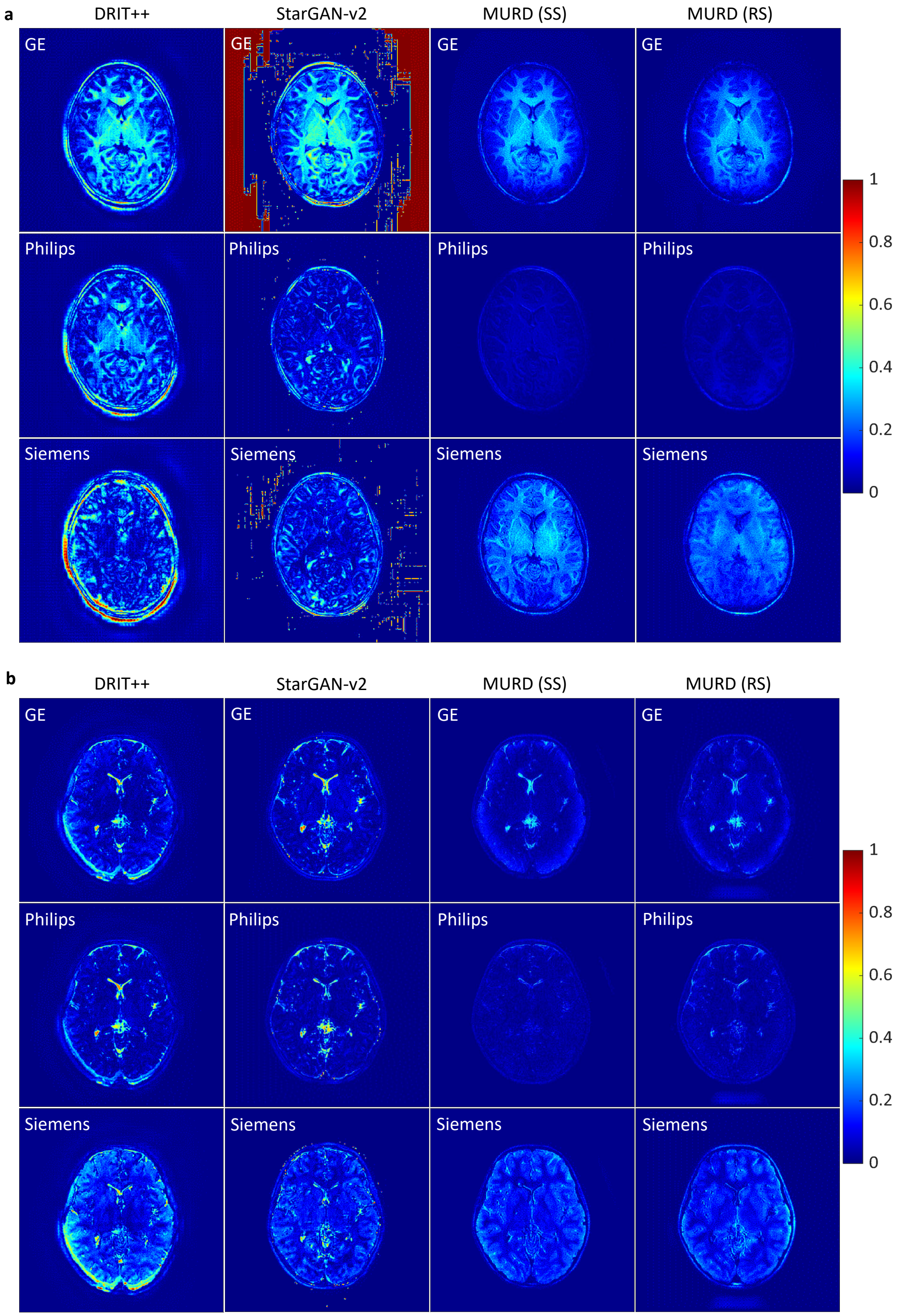

Supplementary Figure 8: Difference maps of Harmonized Philips Images and Corresponding Traveling Phanthom Images. Difference maps of, **a**, T1-weighted and, **b**, T2-weighted images harmonized using various methods with respect to General Electric (GE), Philips, and Siemens sites. In **a** and **b**, the first to fourth columns are respectively difference maps of harmonized images given by DRIT++, StarGAN-v2, site-specific (SS) MURD, and reference-specific (RS) MURD.

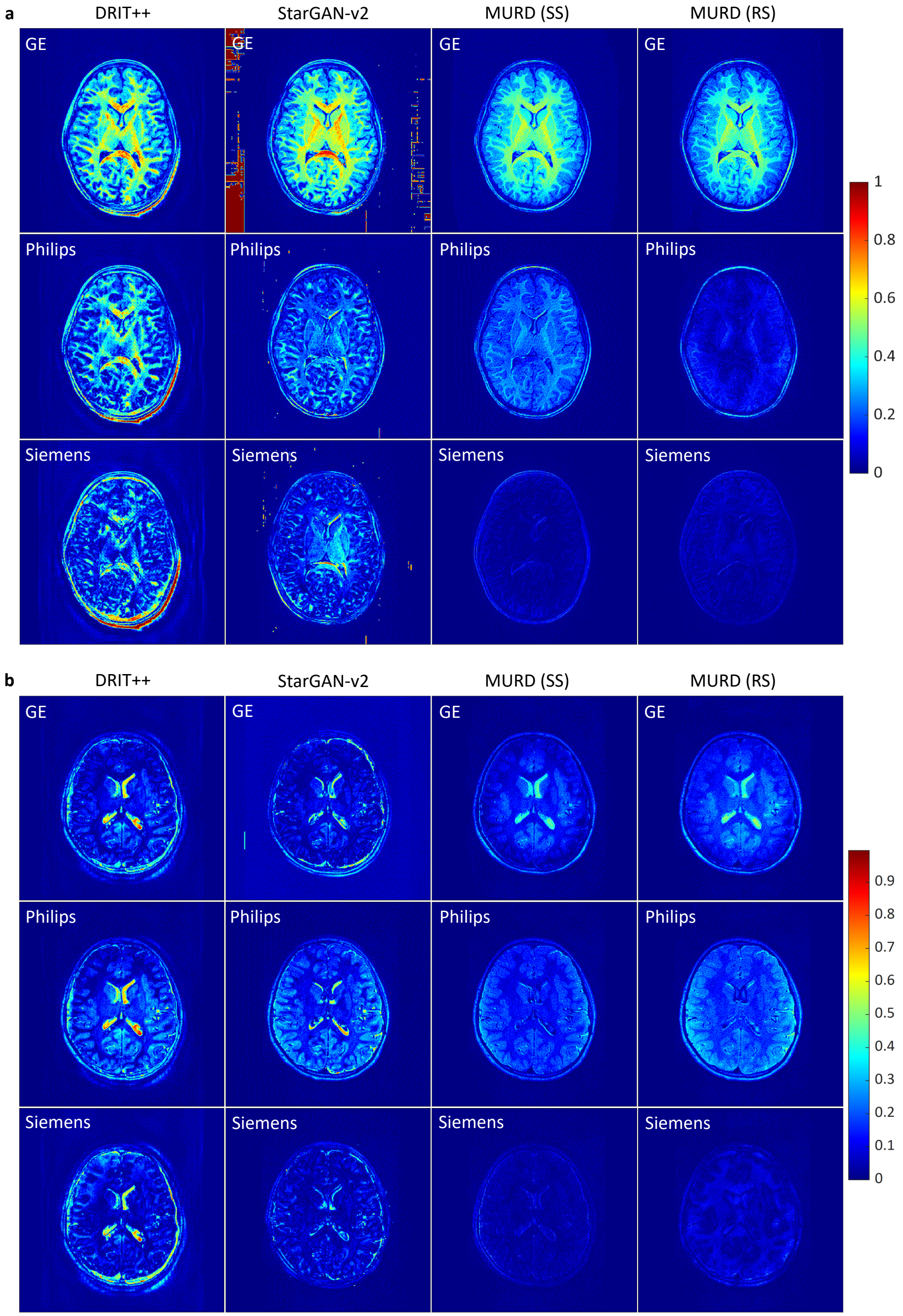

Supplementary Figure 9: Difference maps of Harmonized Siemens Images and Corresponding Traveling Phanthom Images. Difference maps of, **a**, T1-weighted and, **b**, T2-weighted images harmonized using various methods with respect to General Electric (GE), Philips, and Siemens sites. In **a** and **b**, the first to fourth columns are respectively difference maps of harmonized images given by DRIT++, StarGAN-v2, site-specific (SS) MURD, and reference-specific (RS) MURD.

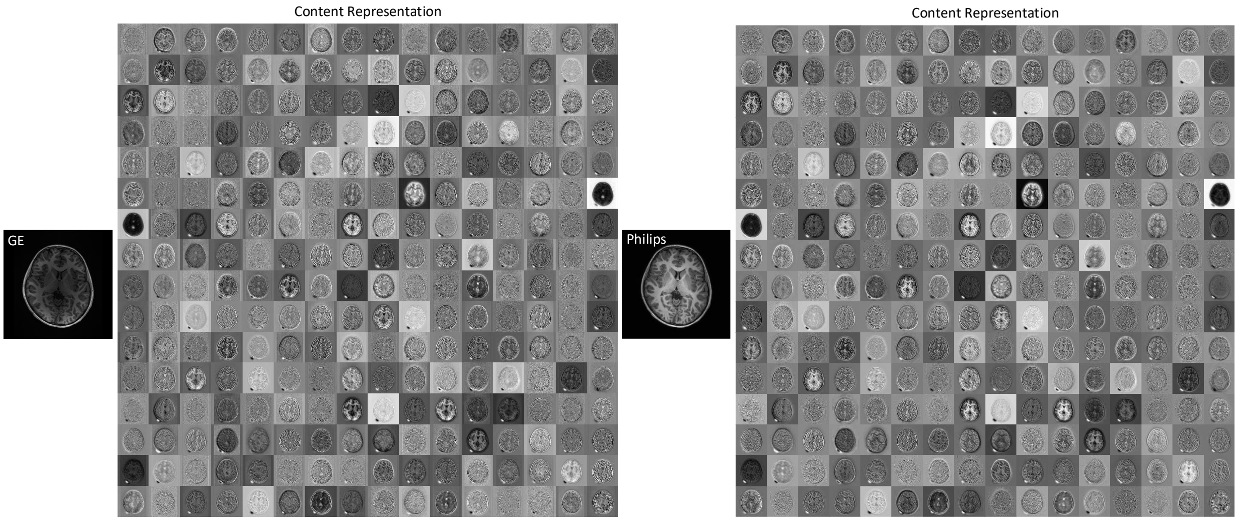

Supplementary Figure 10: Content Representation. The content representations of General Electric (GE) and Philips images of a subject from the traveling human phantom dataset. The representations reflect mostly the anatomical information captured in the MR images and are highly consistent across scanners.

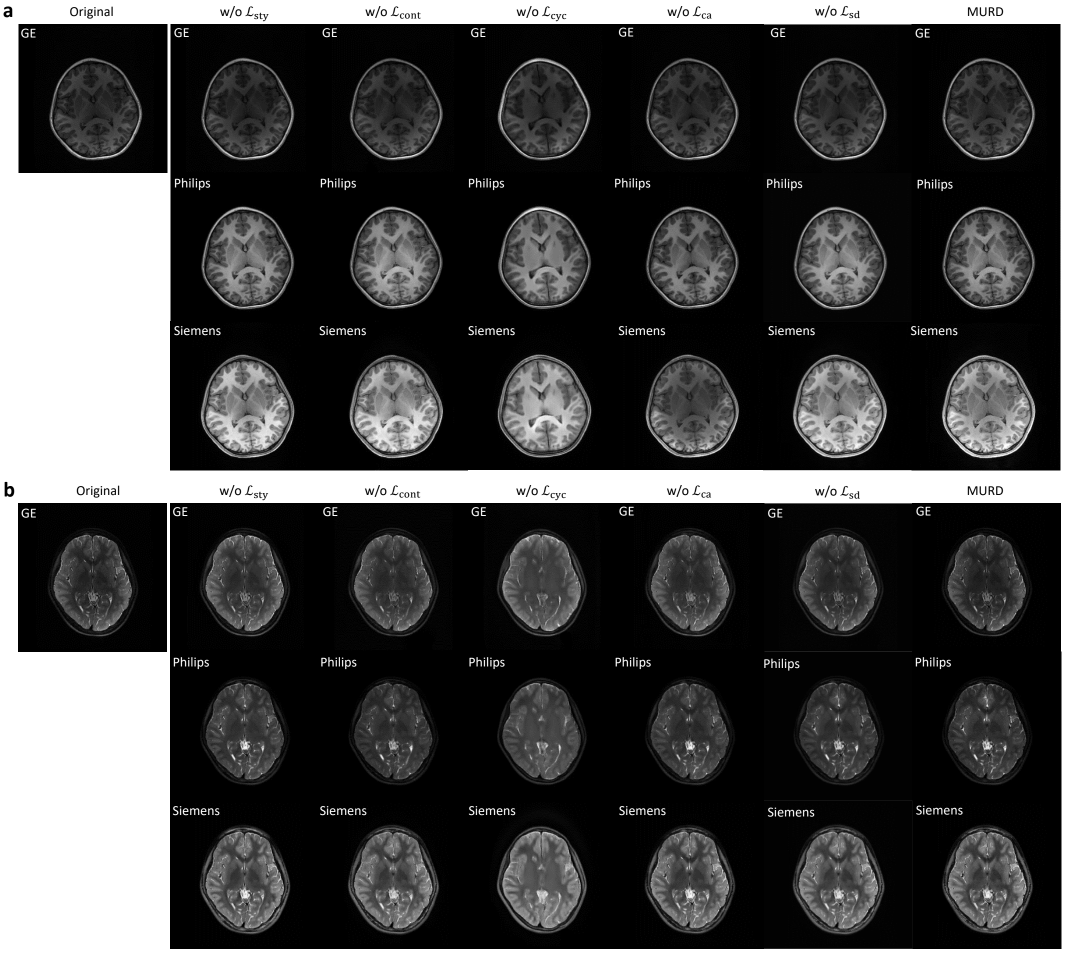

Supplementary Figure 11: Harmonization of General Electric (GE) Images via MURD with Different Losses. **a**, T1-weighted and, **b**, T2-weighted images harmonized with different losses. In **a** and **b**, the first column is the original GE image, the second to seventh columns are respectively the harmonized images using MURD without $\mathcal{L}_{\text{sty}}$, $\mathcal{L}_{\text{cont}}$, $\mathcal{L}_{\text{cyc}}$, $\mathcal{L}_{\text{ca}}$, and with all losses (i.e., MURD).

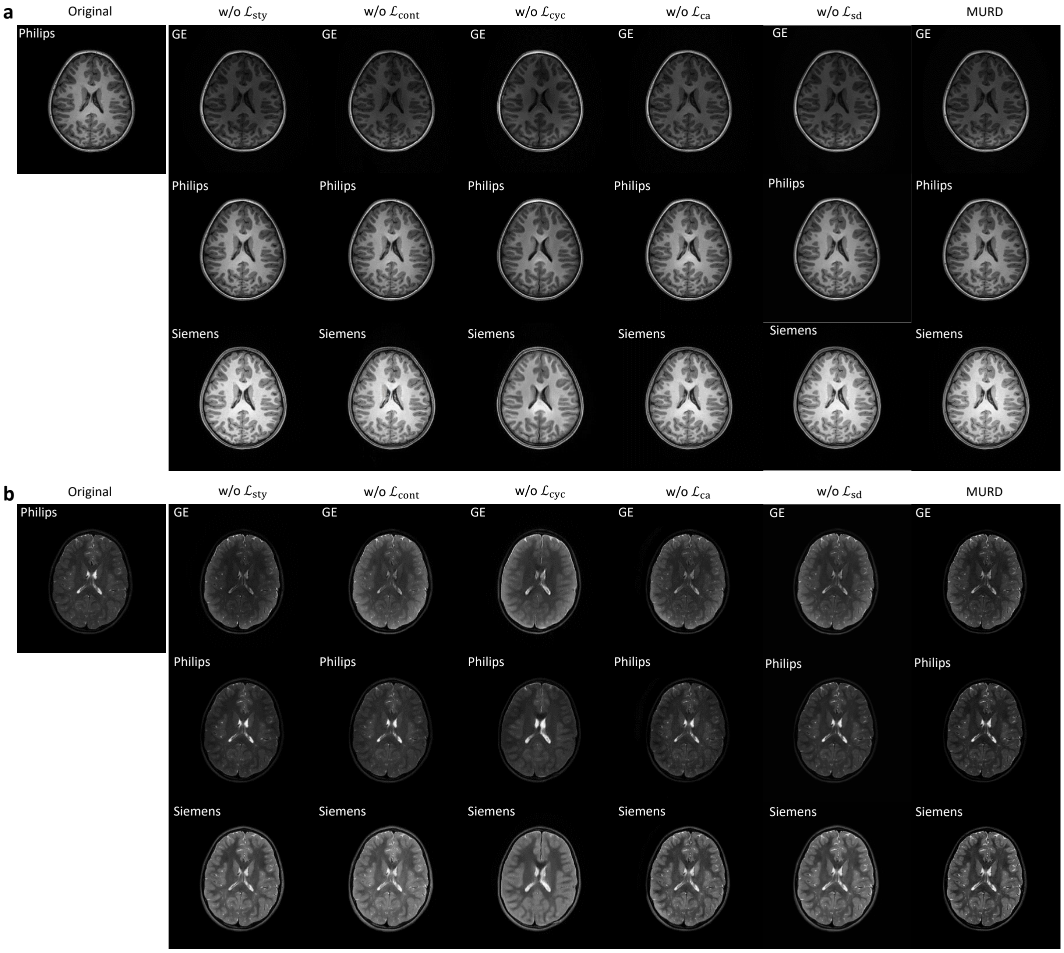

Supplementary Figure 12: Harmonization of Philips Images via MURD with Different Losses. a, T1-weighted and, **b**, T2-weighted images harmonized with different losses. In **a** and **b**, the first column is the original Philips image, the second to seventh columns are respectively the harmonized images using MURD without $\mathcal{L}_{\text{sty}}$, $\mathcal{L}_{\text{cont}}$, $\mathcal{L}_{\text{cyc}}$, $\mathcal{L}_{\text{ca}}$, and with all losses (i.e., MURD).

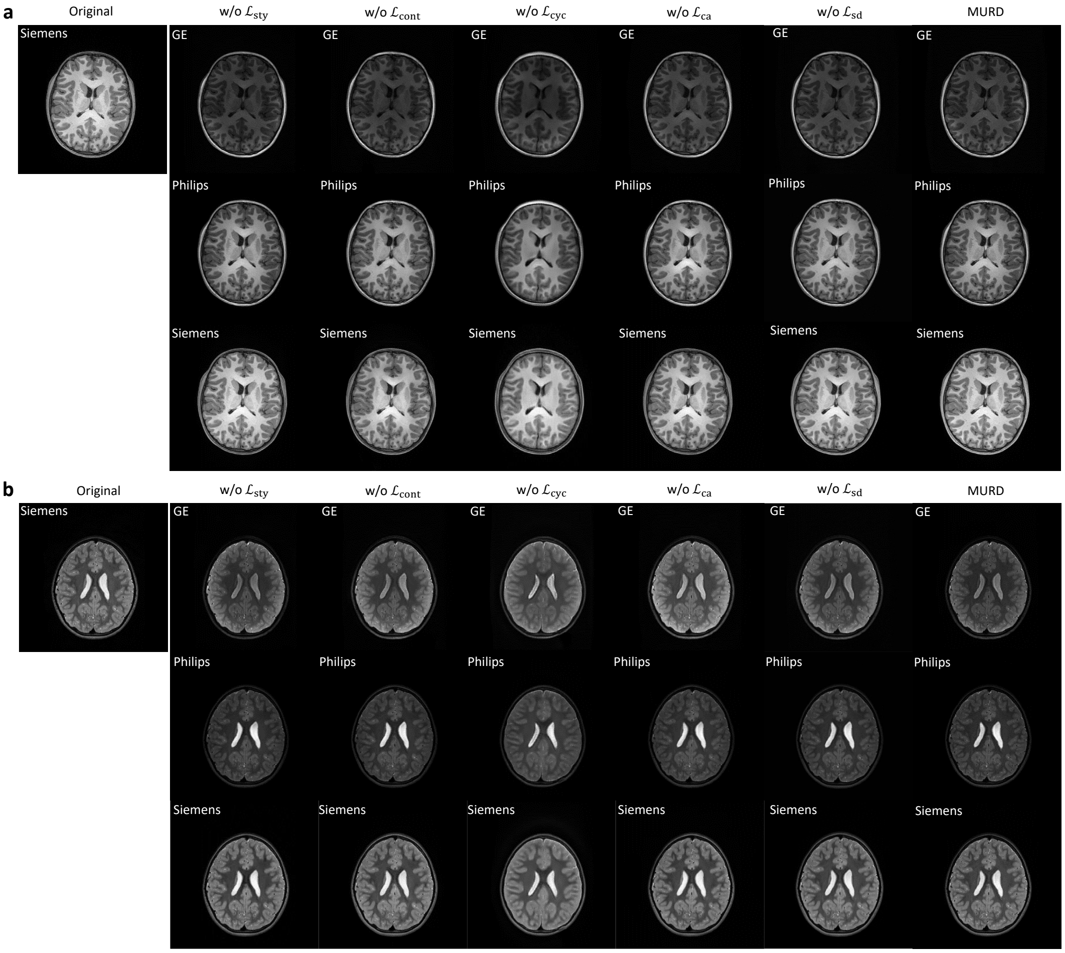

Supplementary Figure 13: Harmonization of Siemens Images via MURD with Different Losses. a, T1-weighted and, **b**, T2-weighted images harmonized with different losses. In **a** and **b**, the first column is the original Siemens image, the second to seventh columns are respectively the harmonized images using MURD without $\mathcal{L}_{sty}$, $\mathcal{L}_{cont}$, $\mathcal{L}_{cyc}$, $\mathcal{L}_{ca}$, and with all losses (i.e., MURD).

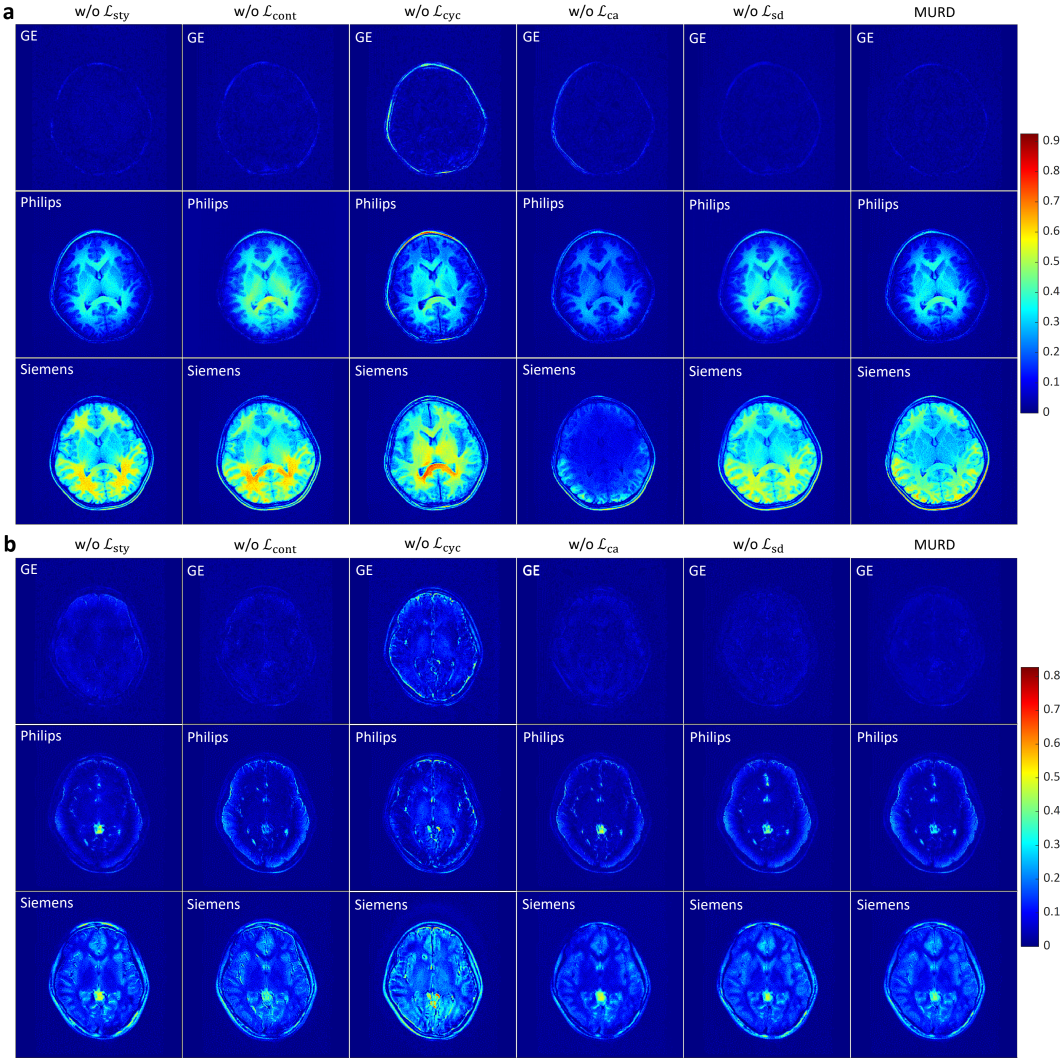

Supplementary Figure 14: Difference Maps of Harmonized General Electric (GE) Images and Corresponding Travelling Phanthom Images via MURD with Different Losses. Difference maps of, **a**, T1-weighted and, **b**, T2-weighted images harmonized with different losses. In **a** and **b**, the first to sixth columns are respectively the difference maps of harmonized images using MURD without $\mathcal{L}_{sty}$, $\mathcal{L}_{cont}$, $\mathcal{L}_{cyc}$, $\mathcal{L}_{ca}$, and with all losses (i.e., MURD).

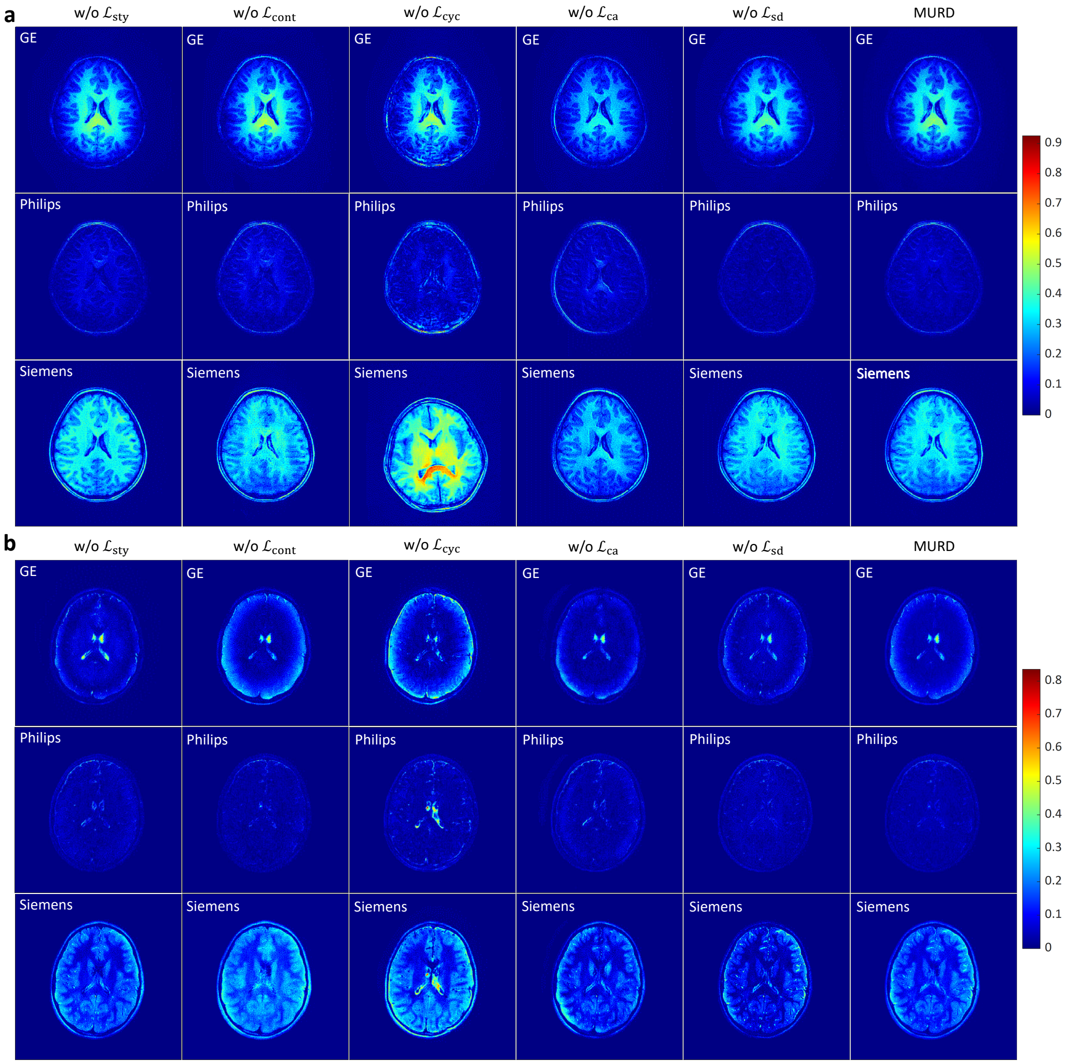

Supplementary Figure 15: Difference Maps of Harmonized Philips Images and Corresponding Travelling Phanthom Images via MURD with Different Losses. Difference maps of, **a**, T1-weighted and, **b**, T2-weighted images harmonized with different losses. In **a** and **b**, the first to sixth columns are respectively the difference maps of harmonized images using MURD without $\mathcal{L}_{\mathrm{sty}}$, $\mathcal{L}_{\mathrm{cont}}$, $\mathcal{L}_{\mathrm{cyc}}$, $\mathcal{L}_{\mathrm{ca}}$, and with all losses (i.e., MURD).

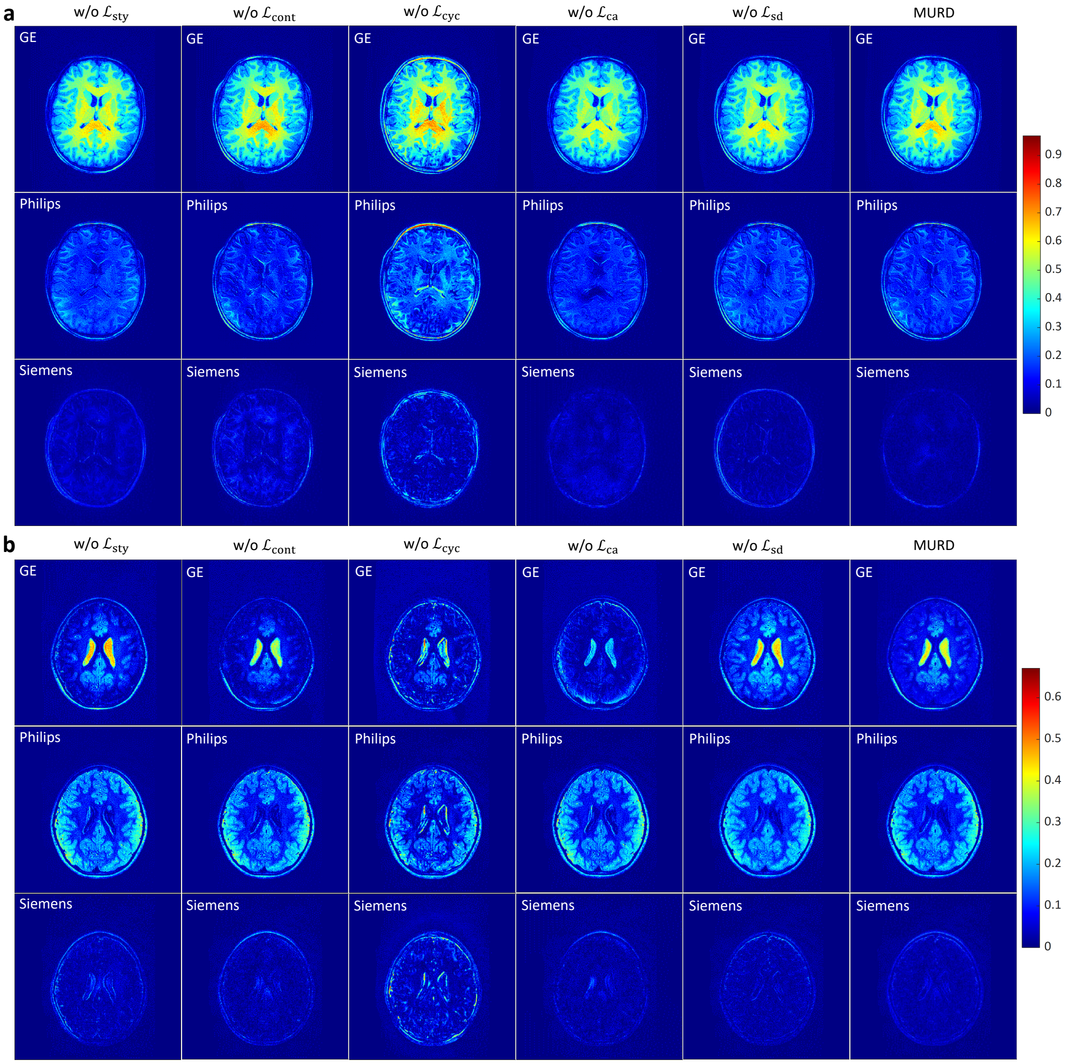

Supplementary Figure 16: Difference Maps of Harmonized Siemens Images and Corresponding Travelling Phantom Images via MURD with Different Losses. Difference maps of, **a**, T1-weighted and, **b**, T2-weighted images harmonized with different losses. In **a** and **b**, the first to sixth columns are respectively the difference maps of harmonized images using MURD without $\mathcal{L}_{\text{sty}}$, $\mathcal{L}_{\text{cont}}$, $\mathcal{L}_{\text{cyc}}$, $\mathcal{L}_{\text{ca}}$, and with all losses (i.e., MURD).

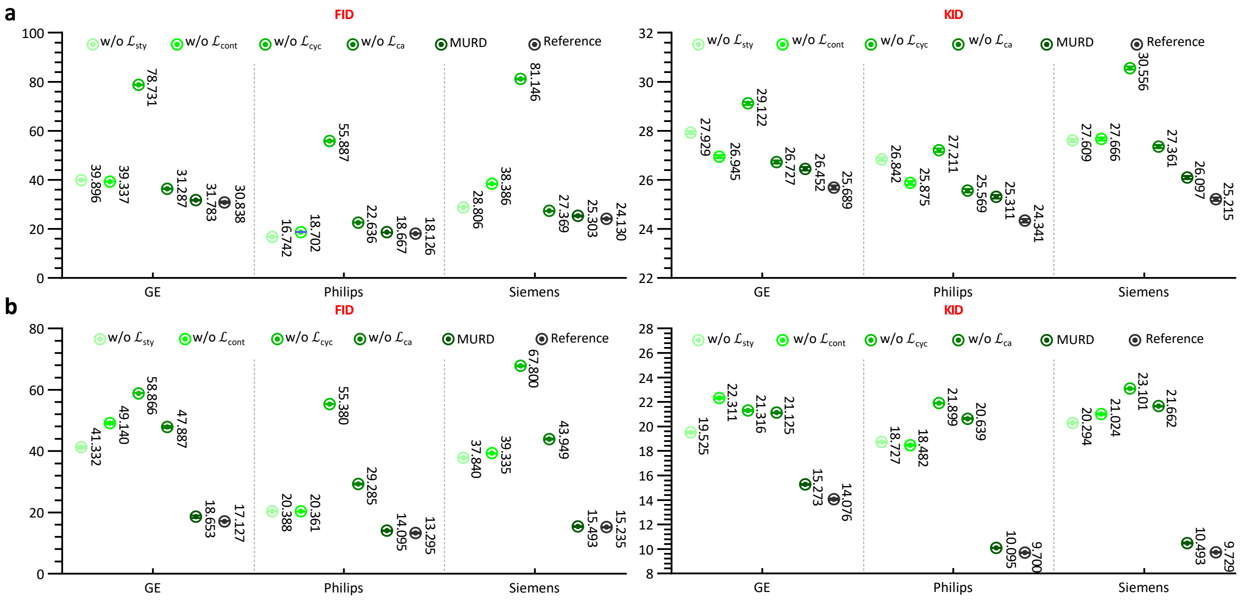

Supplementary Figure 17: Numerical Evaluation of MURD with Different Losses. FID and KID evaluation of, **a**, T1-weighted images ($n = 600$ slices) and, **b**, T2-weighted images ($n = 600$ slices) harmonized without $\mathcal{L}_{sty}$, $\mathcal{L}_{cont}$, $\mathcal{L}_{cyc}$, $\mathcal{L}_{ca}$, and with all losses (i.e., MURD). Bullseyes show the means and error bars show the standard errors on the means. MURD achieves the best performance for all metrics.

Supplementary References

1. Ulyanov, D., Vedaldi, A. & Lempitsky, V. Improved texture networks: maximizing quality and diversity in feed-forward stylization and texture synthesis. In *Proceedings of IEEE Conference on Computer Vision and Pattern Recognition (CVPR)*, 6924–6932 (2017).
2. He, Q., Shiee, N., Reich, D. S., Calabresi, P. A. & Pham, D. L. Intensity standardization of longitudinal images using 4d clustering. In *Proceedings of International Symposium on Biomedical Imaging* (2013).
3. Udupa, L. G. N. J. K. On standardizing the mr image intensity scale. *Magenetic Resonance in Medicine* **42**, 1072–1081 (1999).
4. Shah, M. *et al.* Evaluating intensity normalization on MRIs of human brain with multiple sclerosis. *Medical Image Analysis* **15**, 267–282 (2011).
5. Shinohara, R. T. *et al.* Statistical normalization techniques for magnetic resonance imaging. *NeuroImage: Clinical* **6**, 9–19 (2014).
6. Wrobel, J. *et al.* Intensity warping for multisite MRI harmonization. *NeuroImage* **223**, 117242 (2020).
7. Fortin, J.-P., Sweeney, E. M., Muschelli, J., Crainiceanu, C. M. & Shinohara, R. T. Removing inter-subject technical variability in magnetic resonance imaging studies. *NeuroImage* **132**, 198–212 (2016).
8. Fortin, J.-P. *et al.* Harmonization of multi-site diffusion tensor imaging data. *NeuroImage* **161**, 149–170 (2017).
9. Fortin, J.-P. *et al.* Harmonization of cortical thickness measurements across scanners and sites. *NeuroImage* **167**, 104–120 (2018).
10. Jog, A., Carass, A., Roy, S., Pham, D. L. & Prince, J. L. Random forest regression for magnetic resonance image synthesis. *Medical Image Analysis* **35**, 475–488 (2017).
11. Garcia-Dias, R. *et al.* Neuroharmony: A new tool for harmonizing volumetric MRI data from unseen scanners. *NeuroImage* **220**, 117127 (2020).
12. Dewey, B. E. *et al.* DeepHarmony: A deep learning approach to contrast harmonization across scanner changes. *Magnetic Resonance Imaging* **64**, 160–170 (2019).
13. Dewey, B. E. *et al.* A disentangled latent space for cross-site MRI harmonization. In *Proceedings of International Conference on Medical Image Computing and Computer-Assisted Intervention (MICCAI)*, 720–729 (Springer International Publishing, 2020).
14. Dinsdale, N. K., Jenkinson, M. & Namburete, A. I. Deep learning-based unlearning of dataset bias for MRI harmonisation and confound removal. *NeuroImage* **228**, 117689 (2021).
15. Liu, M. *et al.* Style transfer using generative adversarial networks for multi-site MRI harmonization. *bioRxiv* (2021).
16. Bashyam, V. M. *et al.* Deep generative medical image harmonization for improving cross-site generalization in deep learning predictors. *Journal of Magnetic Resonance Imaging* **55**, 908–916 (2021).
17. Zuo, L. *et al.* Unsupervised MR harmonization by learning disentangled representations using information bottleneck theory. *NeuroImage* **243**, 118569 (2021).
18. Lee, H.-Y. *et al.* DRIT++: Diverse image-to-image translation via disentangled representations. *International Journal of Computer Vision* **128**, 2402–2417 (2020).
19. Choi, Y., Uh, Y., Yoo, J. & Ha, J.-W. StarGAN v2: Diverse image synthesis for multiple domains. In *Proceedings of the IEEE/CVF Conference on Computer Vision and Pattern Recognition (CVPR)* (2020).
